# Wen-Shen-Tong-Luo-Zhi-Tong Decoction alleviates bone loss in aged mice by suppressing LONP1-mediated macrophage senescence

**DOI:** 10.1080/13880209.2025.2537125

**Published:** 2025-07-28

**Authors:** Qinfeng Zhou, Kaixuan Wang, Cong Wang, Xiaoxian Sun, Lining Wang, Jie Sun, Yalan Pan, Muzhe Li, Zitong Zhao, Shijie Zhou, Qing Wang, Yafeng Zhang, Yong Ma, Yang Guo

**Affiliations:** ^a^ Laboratory of New Techniques of Restoration & Reconstruction of Orthopedics and Traumatology, Nanjing University of Chinese Medicine, Nanjing, China; ^b^ Affiliated Hospital of Nanjing University of Chinese Medicine, Nanjing, China; ^c^ Department of Laboratory Medicine, Zhangjiagang TCM Hospital Affiliated to Nanjing University of Chinese Medicine, Suzhou, China; ^d^ School of Integrative Medicine, Nanjing University of Chinese Medicine, Nanjing, China; ^e^ Jiangsu CM Clinical Innovation Center of Degenerative Bone & Joint Disease, Wuxi TCM Hospital Affiliated to Nanjing University of Chinese Medicine, Wuxi, China; ^f^ TCM Nursing Intervention Key Laboratory of Chronic Disease, Nanjing University of Chinese Medicine, Nanjing, China; ^g^ Yancheng TCM Hospital Affiliated to Nanjing University of Chinese Medicine, Yancheng, China

**Keywords:** Wen-Shen-Tong-Luo-Zhi-Tong Decoction, LONP1, macrophage senescence, cGAS/STING, BMSC osteogenesis

## Abstract

**Context:**

Aging leads to senile osteoporosis (SOP), marked by bone loss and increased fracture risk. Macrophages, as active immune cells in bone tissue, play an important role in osteogenic differentiation of bone marrow mesenchymal stem cells (BMSCs) during aging. Wen-Shen-Tong-Luo-Zhi-Tong Decoction (WSTLZTD), a traditional Chinese herbal formula, has been clinically validated for its efficacy in treating SOP. However, the specific mechanisms by which WSTLZTD exerts its anti-SOP effects—particularly through modulating macrophage senescence—remain unclear.

**Objective:**

The study aims to elucidate the role of WSTLZTD in macrophage senescence and SOP.

**Materials and methods:**

Aged mice received low, medium, high-dose WSTLZTD. Bone loss was evaluated via micro-computed tomography, hematoxylin and eosin staining and osteocalcin, tartrate-resistant acid phosphatase marker analysis. Macrophage senescence detection (β-galactosidase staining, p16, p21) and molecular mechanisms by Western blot, immunohistochemistry, immunofluorescence method were investigated. Macrophage-conditioned medium’s effects on BMSC osteogenesis and mitochondrial function were assessed through alkaline phosphatase, Alizarin Red S staining, reactive oxygen species and JC-1 mitochondrial membrane potential (ΔΨm) assays.

**Results:**

*In vivo* experiments demonstrated that WSTLZTD effectively ameliorated macrophage senescence and osteoporosis in naturally aged mice. Mechanistically, high-dose WSTLZTD attenuated senescence in bone marrow-derived macrophages by mediating LONP1, concurrently suppressing the cyclic GMP-AMP synthase (cGAS)/STING signaling pathway in BMSCs, thereby enhancing osteogenic differentiation of BMSCs. *In vitro* studies further confirmed that WSTLZTD-containing serum attenuated the senescent phenotype of macrophages. Notably, the LONP1 inhibitor, LONP1-IN-2, was found to diminish the anti-senescence effects of WSTLZTD on macrophages and BMSC osteogenesis.

**Discussion and conclusion:**

WSTLZTD potentially modulate macrophage senescence via LONP1, which subsequently suppresses the activation of the cGAS/STING pathway in BMSCs, ultimately promoting their osteogenic differentiation and ameliorating osteoporosis.

## Introduction

Aging is a degenerative process influenced by both intrinsic and extrinsic environmental factors, characterized by the gradual decline in structural integrity and physiological functions. Senile osteoporosis (SOP), a musculoskeletal manifestation of aging, is a prevalent skeletal disorder marked by reduced bone mass, deterioration of bone microstructure and increased fracture risk in the elderly population (Reid and Billington 2022). Bisphosphonates, such as alendronate (ALN), remain one of the most widely prescribed therapeutic agents; however, their long-term application is often limited by adverse effects (Yilmaz et al. 2012). Thus, identifying safer treatment strategies and further exploring the mechanisms underlying SOP pathogenesis are crucial to address current therapeutic limitations.

Immunosenescence, defined by phenotypic and functional alterations in immune cells, adversely affects bone cellular activities and metabolic equilibrium, thereby intensifying the disparity between bone resorption and formation and increasing susceptibility to osteoporosis (Pappert et al. 2023). The concept of ‘osteoimmunology’, first proposed in *Nature* in 2000, highlights the critical role of immune dysregulation in bone metabolism disorders (Arron and Choi 2000). Macrophages, a heterogeneous population of innate immune cells, polarize into pro-inflammatory M1 or anti-inflammatory M2 subtypes. Aging drives macrophages toward M1 polarization, accompanied by upregulated senescence-associated secretory phenotype (SASP), which disrupts bone metabolism and suppresses osteogenesis (Bai et al. 2023). Accumulation of senescent macrophages in the bone marrow with aging correlates with reduced bone turnover rate and increased skeletal fragility (Cheng et al. 2024). Bone marrow mesenchymal stem cells (BMSCs), multipotent progenitors essential for osteoblast differentiation and bone homeostasis, exhibit functional impairment when exposed to senescent macrophages, contributing to osteoporosis pathogenesis. Li CJ et al. (2021) observed elevated senescent macrophages and concomitant declines in BMSC osteogenic capacity and bone mass in aged murine bone marrow. Furthermore, Bai et al. demonstrated that co-culture of senescent macrophages with healthy BMSCs significantly reduced alkaline phosphatase (ALP) activity and Alizarin Red S (ARS)-positive mineralization, whereas senescence inhibition restored BMSC osteogenic potential (Bai et al. 2023). These findings underscore macrophage senescence as a pivotal driver of BMSC dysfunction and SOP progression.

Wen-Shen-Tong-Luo-Zhi-Tong Decoction (WSTLZTD), a traditional Chinese herbal formula developed by Professor Yong Ma’s team, has demonstrated clinical efficacy in treating osteoporosis and related symptoms through 15 years of practice (Ma et al. 2010). Clinical studies have shown that WSTLZTD significantly improves outcomes in osteoporosis patients, including symptom relief, enhanced bone mineral density (BMD) and improved biochemical markers of bone metabolism, with no notable adverse effects such as systemic toxicity or abnormal blood, urine, fecal or electrocardiogram results (Zheng et al. 2016). While Zhou et al. (2020) reported that combining WSTLZTD with conventional treatments like calcium and vitamin D resulted in more significant improvements in BMD, pain scores (Visual Analog Scale) and Traditional Chinese Medicine (TCM) symptom scores compared to the control group. Additionally, our preclinical studies reveal that WSTLZTD exerts multi-target therapeutic effects in various osteoporosis models. For instance, it enhances testosterone secretion, revitalizes mitochondrial energy metabolism and promotes osteogenic differentiation of senescent BMSCs in SAMP6 mice (Li M et al. 2025). Notably, our recent work found that *in vitro* hydrogen peroxide (H_2_O_2_)-induced senescent RAW264.7 macrophages can affect the osteogenesis of BMSCs (Li M et al. 2024). Although these findings provide preliminary insights, the anti-osteoporotic effects of WSTLZTD in naturally aged mice have not yet been validated. More importantly, the specific molecules targeted and regulated by WSTLZTD in endogenous senescent macrophages, as well as the precise mechanisms through which these molecules mediate their effects, remain to be elucidated.

In the current research, we demonstrated that WSTLZTD exhibits a bone loss-suppressive effect, potentially through the suppression of LONP1-mediated macrophage senescence and the cyclic GMP-AMP synthase (cGAS)/STING pathway in BMSCs. These findings further substantiate the potential of WSTLZTD as a therapeutic agent against SOP.

## Methods

### Experimental animals

Three-month-old male C57BL/6J mice and 16-month-old male C57BL/6J mice were purchased from Jiangsu Huachuang Co., Ltd. (License No.: SCXK (Jiangsu) 2020-0009). Eight-week-old male Sprague-Dawley (SD) rats and 6-day-old male C57BL/6J mice were obtained from Qinglongshan Animal Breeding Facility (License No.: SCXK (Jiangsu) 2024-0001). All animals were housed in a controlled environment with a 12-h light/dark cycle, at a temperature of 22 ± 2 °C and humidity maintained at 50–60%. The animals were provided with unrestricted access to standard laboratory rodent chow and fresh drinking water. All animal experiments were approved by the Institutional Animal Care and Use Committee of Nanjing University of Chinese Medicine (Approval No.: 202403A068) and conducted in accordance with the Guide for the Care and Use of Laboratory Animals and the ARRIVE guidelines (https://arriveguidelines.org/).

### Preparation and phytochemical profiling of WSTLZTD

WSTLZTD comprises 15 herbs, as indicated in Table 1. The batch numbers of the 15 herbs are as follows: Aconiti Lateralis Radix Praeparata: 250208; Corni Fructus: 2308052; Drynariae Rhizoma: 2302004; Epimedii Folium: 240501; Cnidii Fructus: 240703; Cibotii rhizoma: 2408110; Coicis Semen: 2302017; Atractylodis Macrocephalae Rhizoma: 2410027; Notopterygii rhizoma et radix: 240901; Angelicae Pubescentis Radix: 2302009; Asari Radix et Rhizoma: 2306034; Gastrodiae Rhizoma: 240701; Glycyrrhizae Radix et Rhizoma: 240801; Astragali Radix: 240601; Paeoniae Radix Alba: 2303158. Crude herbal materials were purchased from the National TCM Experts Clinic, Nanjing University of Chinese Medicine and authenticated by Prof. Y. Guo, Nanjing University of Chinese Medicine. Voucher specimens with specific storage code were well-deposited at Nanjing university of Chinese medicine. Herbs were soaked in distilled water at a 10:1 (v/w) ratio for 0.5 h, heated to boiling and decocted for 1 h, and residues were re-extracted with distilled water (8:1 v/w) for another hour. The combined decoctions were filtered, concentrated and stored at −80 °C.

**Table 1.** Composition of WSTLZTD.

Botanical name	Chinese name	Plant part used	Dosage used
Aconiti Lateralis Radix Praeparata	Fuzi	Root of *Aconitum carmichaelii Debx*	8 g
Corni Fructus	Shanzhuyu	Ripe fruit of *Cornus officinalis Sieb. et Zucc*	10 g
Drynariae Rhizoma	Gusuibu	Rhizome of *Drynaria fortunei (Kunze)J.Sm*	30 g
Epimedii Folium	Yinyanghuo	Leaves of *Epimedium brevicornu Maxim*	10 g
Cnidii Fructus	Shechuangzi	Fruit of *Cnidium monnieri (L.) Cuss*	6 g
Cibotii rhizome	Gouji	Rhizome of *Cibotium barometz (L.) J.Sm*	10 g
Coicis Semen	Yiyiren	Seeds of *Coix lachryma-jobi var. Mayuen*	15 g
Atractylodis Macrocephalae Rhizoma	Baizhu	Rhizome of *Atractylodes macrocephala Koidz*	10 g
Notopterygii rhizoma et radix	Qianghuo	Rhizome and root of *Notopterygium incisum Ting ex H. T. Chang*	10 g
Angelicae Pubescentis Radix	Duhuo	Root of *Angelica pubescens Maxim.f. biserrata Shan et Yuan*	10 g
Asari Radix et Rhizoma	Xixin	Rhizome and root of *Asarum heterotropoides Fr. Schmidt* var. *mandshuricum (Maxim.) Kitag*	3 g
Gastrodiae Rhizoma	Tianma	Stem of *Gastrodia elata Bl*	6 g
Glycyrrhizae Radix et Rhizoma	Gancao	Rhizome and root of *Glycyrrhiza uralensis Fisch*	6 g
Astragali Radix	Huangqi	Root of *Astragalus membranaceus (Fisch.) Bge.var. mongholicus (Bge.)Hsiao*	15 g
Paeoniae Radix Alba	Baishao	Root of *Paeonia lactiflora Pall*	15 g

Liquid chromatography tandem mass spectrometry (LC–MS/MS) analysis was conducted on a Q-Exactive mass spectrometer (Thermo Fisher Scientific) coupled with an ACQUITY UPLC I-Class Plus system (Waters) using heated ESI in both positive and negative modes. Chromatographic separation was achieved with an ACQUITY UPLC HSS T3 column (2.1 × 100 mm, 1.8 μm) at 45 °C, with a mobile phase of 0.1% formic acid in water and acetonitrile. The flow rate was 0.35 mL/min. Samples were maintained at 4 °C during analysis. The mass spectrometer operated with *m/z* 100–1200 scan range, 70,000 (MS) and 17,500 (MS/MS) resolutions, stepped collision energies (10/20/40 eV), electrospray voltages at ±3800/3000 V, sheath/auxiliary gas flows 35/8 arb. units, capillary/auxiliary temperatures 320/350 °C and S-lens RF 50.

### Preparation of drug-containing serum in rats

Eight-week-old male SD rats, weighing approximately 280–300 g, were randomly divided into four groups: control group, low-, medium- and high-dose WSTLZTD groups. The therapeutic groups received low-, medium- and high-dose WSTLZTD suspension via oral gavage at 10 mL/kg body weight once daily, while the control group received an equivalent volume of normal saline once daily for seven consecutive days. Two hours after the final administration, blood samples were collected via the abdominal aorta under isoflurane anesthesia. Serum was separated by centrifugation at 3500 r/min for 15 min and stored at −80 °C for further use.

### Experimental groups and interventions

Mice were divided into six groups (*n* = 10/group): Control group: 3-month-old male C57BL/6J mice, weighing approximately 25–30 g, receiving daily saline administration. Model group: 16-month-old male C57BL/6J mice, weighing approximately 30–35 g, receiving daily saline administration. Low-dose WSTLZTD group: 16-month-old male C57BL/6J mice administered 0.105 g/10 g body weight/day WSTLZTD. Medium-dose WSTLZTD group: 16-month-old male C57BL/6J mice administered 0.21 g/10 g body weight/day WSTLZTD. High-dose WSTLZTD group: 16-month-old male C57BL/6J mice administered 0.42 g/10 g body weight/day WSTLZTD (Li M et al. 2025). Positive control: 16-month-old male C57BL/6J mice receiving weekly ALN (9.1 mg/kg body weight) (Fosamax, HJ20160100). Treatments were administered via oral gavage for 10 weeks. Then animals were anesthetized with 2% isoflurane (induction: 5% for 2 min; maintenance: 2%) delivered via a calibrated vaporizer at a fresh gas flow rate of 4 L/min. Anesthesia depth was monitored by absence of pedal withdrawal reflex. Following the attainment of adequate anesthesia, euthanasia was conducted via cervical dislocation in accordance with ethical guidelines, to facilitate further analysis.

### Cell culture

#### Bone marrow-derived macrophages

Bone marrow cells from femurs and tibias of euthanized mice were flushed with RPMI 1640 (Shanghai Yuanpei Biotechnology, L210KJ) containing 50 ng/mL Recombinant Murine M-CSF (PeproTech, 315-02), 10% fetal bovine serum (FBS) (New Zealand, Cytiva, SH30406.05) and 1% penicillin–streptomycin (NCM Biotech, C100C5). Cells (1 × 10^6^/mL) were cultured for 7 days, with medium changes on days 3 and 5. Adherent bone marrow-derived macrophages (BMDMs) were harvested for experiments.

#### BMSCs

Following the euthanasia of 6-day-old male C57BL/6J mice, femurs and tibias were meticulously extracted. The bones were subsequently flushed with sterile PBS using a syringe to generate a single-cell suspension. This suspension was then seeded into α-MEM medium (Gibco, C12571500BT) supplemented with 10% FBS (New Zealand, Cytiva, SH30406.05) and 1% penicillin–streptomycin (NCM Biotech, C100C5) and incubated at 37 °C in a 5% CO_2_ atmosphere. After a 24-h incubation period, the culture medium was replaced to eliminate non-adherent cells. The adherent cells were cultured until they achieved 80–90% confluence, at which point they were passaged for further experimentation. Cells at passages 2–3 were collected and cryopreserved at −80 °C for subsequent experiments. For osteogenic differentiation, BMSCs were cultured in a commercially available osteogenic induction medium (Wuhan Pricella, PD003).

#### RAW 264.7 cells

RAW 264.7 macrophages (Procell, CL-0190) were maintained in high-glucose DMEM (Gibco, 11965092) with 10% FBS (New Zealand, Cytiva, SH30406.05) and 1% penicillin–streptomycin (NCM Biotech, C100C5), then subcultured at a 1:3 ratio every 2 days.

### Group of experiments

#### Micro-computed tomography

The microstructural properties of the distal femur were analyzed using a micro-computed tomography (micro-CT) system (SkyScan 1272, Bruker). Bones were scanned at high resolution (9 μm) with an energy setting of 50 kV and 456 μA. Three-dimensional reconstructions and analyses were performed using NRecon v1.6 and CTAn v1.13.8.1 software (Bruker). The region of interest was defined as 0.1 mm below the growth plate to 5% of the femoral length. Parameters including BMD, bone volume fraction (BV/TV), trabecular number (Tb.N), trabecular thickness (Tb.Th) and trabecular separation (Tb.Sp) were quantified.

#### Hematoxylin and eosin staining

Femoral tissues were fixed in 4% paraformaldehyde (Beyotime, P0099) for 24 h, decalcified in ethylenediaminetetraacetic acid solution (Servicebio, G1105) for 1 month and embedded in paraffin. Sections (5 μm thick) were deparaffinized, rehydrated and stained with hematoxylin and eosin (H&E). Images were captured under a light microscope.

#### Tartrate-resistant acid phosphatase staining

Deparaffinized sections were dehydrated through an ethanol gradient, washed with distilled water and incubated in pre-warmed tartrate-resistant acid phosphatase (TRAP) staining solution (Servicebio, G105) at 37 °C for 20 min. Nuclei were counterstained with hematoxylin, followed by differentiation and bluing steps. Sections were dehydrated, cleared and mounted for microscopic imaging.

#### Immunohistochemistry

Tissue sections were baked at 60 °C for 1 h, deparaffinized in xylene and rehydrated. After antigen retrieval and blocking with 5% Bovine Serum Albumin(BSA) (Beyotime, ST025) sections were incubated overnight at 4 °C with primary antibodies against osteocalcin (OCN) (Proteintech, 16157-1-AP, 1:250), LONP1 (Proteintech, 15440-1-AP, 1:250), cGAS (Proteintech, 29958-1-AP, 1:250) and STING (Proteintech, 19851-1-AP, 1:250). Horseradish Peroxidase (HRP)-conjugated secondary antibody (RecordBio, RCB054) were applied for 20 min at room temperature, followed by DAB visualization. Slides were counterstained with hematoxylin, dehydrated and mounted.

#### Enzyme-Linked immunosorbent assay

Serum levels of ALP, OPG, RUNX2, IL-6 and IL-1β were quantified using commercial enzyme-linked immunosorbent assay kits (Jiangsu Jingmei Biological Technology Co., Ltd., China). The corresponding catalog numbers were: JM-02494M1, JM-02427M1, JM-11597M1, JM-02446M1, JM-02323M1. Fifty-microliter samples or standards and 50 μL biotin-labeled antibodies were added sequentially. After 30 min incubation at 37 °C, plates underwent five washes. HRP-conjugated antibodies were added, incubated for 30 min at 37 °C, followed by substrate addition. Reactions were stopped after 15 min, and absorbance was measured at 450 nm.

#### Tyramide signal amplification immunofluorescence

For tissue sections, deparaffinization, rehydration and antigen retrieval were performed. For cells, fixation (10–30 min), permeabilization with 0.3% Triton X-100 and PBS washing. Samples were blocked with 5% BSA, incubated overnight with primary antibodies against F4/80 (Abcam, Ab300421, 1:250) or cGAS (Proteintech, 29958-1-AP, 1:250) and treated with HRP-conjugated secondary antibody (RecordBio, RCB054). Tyramide signal amplification and 4′,6-Diamidino-2-phenylindoledihydrochloride (DAPI) nuclear staining were performed. For dual labeling, antibodies were eluted, followed by a second round of staining with antibodies against p16 (Abcam, Ab211542, 1:200), p21 (Abcam, Ab188224, 1:250) or STING (Proteintech, 19851-1-AP, 1:250).

#### β-Galactosidase staining

Cells were fixed for 15 min, washed and incubated overnight at 37 °C (CO_2_-free) with X-Gal-containing staining solution (Beyotime, C0602). Senescent cells were identified by blue puncta under a microscope.

#### ALP staining

Cells were fixed with 4% paraformaldehyde (Beyotime, P0099) for 20 min, washed and incubated with BCIP/NBT staining solution (Beyotime, C3206) for 4 h at room temperature. Images were acquired after PBS washing.

#### ARS staining

BMSCs were fixed and stained with ARS staining Kit (Beyotime, C0148S) for 2 h. Calcium nodules were visualized and quantified microscopically.

#### Reactive oxygen species staining in BMSCs

Intracellular reactive oxygen species (ROS) levels were detected using a ROS Assay Kit (Beyotime, S0033S). The DCFH-DA fluorescent probe was diluted 1:1000 in serum-free culture medium to a final concentration of 10 μM. Culture medium was removed, and cells were incubated with sufficient diluted DCFH-DA to fully cover the monolayer (≥1 mL per well in a six-well plate) at 37 °C for 45 min. Cells were washed three times with serum-free medium to remove extracellular DCFH-DA. For positive controls, Rosup (50 mg/mL) was diluted 1:1000 and applied to cells for 20–30 min to induce ROS overproduction. Nuclei were counterstained with Hoechst 33342 (Beyotime, C1028) for 5 min, and images were acquired using fluorescence microscopy.

#### JC-1 staining in BMSCs

Culture medium was aspirated, and cells were washed once with PBS. 1 mL JC-1 staining solution (Beyotime, C2006) was added to each well and incubated at 37 °C for 30 min. After washing twice with 1× assay buffer, 2 mL of fresh culture medium was added. Mitochondrial membrane potential (ΔΨm) was visualized using fluorescence microscopy. CCCP (10 μM) was used as a positive control and applied to cells for 20 min prior to staining.

#### Network pharmacology

The potential target genes of 22 active ingredient combinations screened from WSTLZTD were predicted using Swiss Target Prediction database (http://www.swisstargetprediction.ch). Disease-related targets were retrieved from the GeneCards (https://www.Genecards.org) and OMIM (https://omim.org) databases, using keywords ‘senile osteoporosis’ and ‘osteoporosis’, followed by removal of duplicate targets. Drug targets and disease targets were imported into the Venny 2.1.0 platform to identify overlapping targets and generate a Venn diagram. The interactions among drugs, components, overlapping targets and diseases were visualized by constructing networks using Cytoscape 3.9.1 and Cytoscape 3.7.0 software. The protein–protein interaction (PPI) network of overlapping targets was analyzed using the STRING database(https://string-db.org). Gene Ontology (GO) functional enrichment analysis and Kyoto Encyclopedia of Genes and Genomes (KEGG) pathway enrichment analysis were performed on the overlapping targets via the DAVID database (https://davidbioinformatics.nih.gov).

#### Molecular docking

Molecular docking is a powerful method for identifying potential therapeutic compounds and predicting ligand-target interactions at the molecular level. In this study, the molecular structure files of key compounds contained in WSTLZTD were retrieved from the PubChem database (https://pubchem.ncbi.nlm.nih.gov). The structural file of the LONP1 receptor protein was obtained from the UniProt database. The AutoDockTools software was employed to modify the LONP1 receptor protein by adding hydrogen atoms and balancing charges, followed by converting both the receptor protein and individual ligand molecules into the pdbqt format. Molecular docking between the receptor protein and ligand molecules was performed using AutoDock Vina 1.1.2, and the docking results were analyzed using Discover Studio. The visualization of docking results was generated with PyMOL.

#### Western blot

Proteins from cells or tibial tissues were extracted using RadioImmunoPrecipitation Assay (RIPA) lysis buffer (Beyotime, P0013K) with PMSF (Beyotime, ST506). Protein concentration was determined with a Bicinchoninic Acid (Assay) (BCA) kit (Beyotime, P0010). Samples were separated by SDS-PAGE, transferred to Polyvinylidene difluoride (PVDF) membranes (Merck Millipore, ISEQ00010) and blocked with 5% skim milk. Membranes were incubated overnight with primary antibodies: anti-LONP1 (Cell Signaling Technology, #P36776, 1:1000), anti-cGAS (Cell Signaling Technology, #Q8C6L5, 1:1000), anti-STING (Proteintech, 19851-1-AP, 1:10,000) and anti-Glyceraldehyde-3-phosphate dehydrogenase (GAPDH) (Proteintech, 10494-1-AP, 1:5000). HRP-conjugated secondary antibodies (Beyotime, A0208, 1:2000) and Enhanced chemiluminescence (ECL) substrate were used for signal detection. Band intensities were quantified using ImageJ.

### Statistical analysis

Data analyses were performed using GraphPad Prism 9.5.0 (GraphPad Software, San Diego, CA, USA). Intergroup differences were analyzed by one-way analysis of variance followed by Tukey’s *post hoc* test for multiple comparisons. Continuous data are presented as mean ± standard deviation. A probability value of *p* < 0.05 was considered statistically significant. The letter ‘*n*’ denotes the number of individual specimens or independent experiments. For each experiment, the minimum number of biological samples used was *n* = 3. The specific number of biological replicates for each experiment is provided in the figure legend.

## Results

### Qualitative analysis of chemical components in WSTLZTD

**Figure 1.**
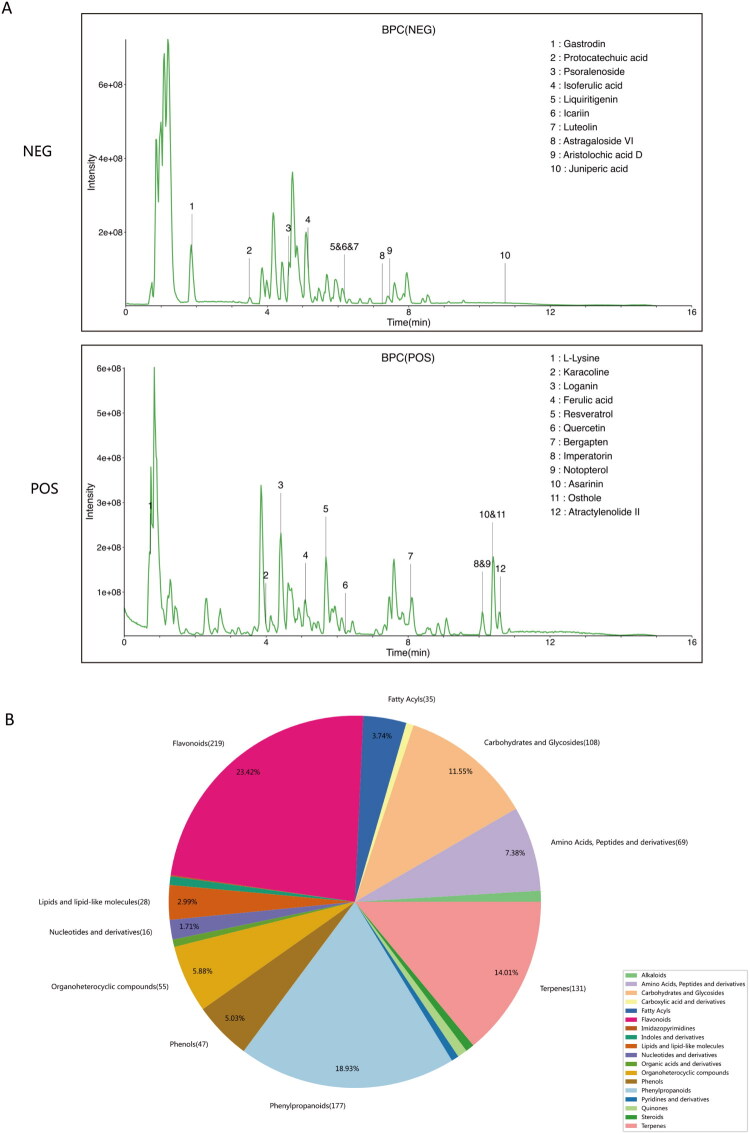
Chemical profiling of WSTLZTD using LC–MS. (A) BPC in negative-ion mode and positive-ion mode. (B) Quantitative distribution of major chemical constituents in WSTLZTD.

Following data processing, qualitative analysis of chemical compounds was conducted using an ultra-performance liquid chromatography–high resolution mass spectrometry platform. The analysis utilized databases including self-built library LuMet-TCM (containing 5000+ TCM component standards established with Shanghai OE Biotech reference materials) and the public HerbDB database. Figure 1(A) presents the base peak chromatogram (BPC) demonstrating major compound identification. Supplementary Data Figure S1 presents the extracted ion chromatograms and secondary mass spectrometry data for the principal bioactive compounds identified in the analysis depicted in Figure 1(A). Figure 1(B) summarizes the primary chemical constituents of WSTLZTD, encompassing phenylpropanoids, flavonoids, terpenoids, fatty acyl derivatives, glycosides, etc., which aligns with the classification of pharmacologically active substances identified in our previous studies (Li M et al. 2025).

### WSTLZTD ameliorates bone loss in SOP mice

**Figure 2.**
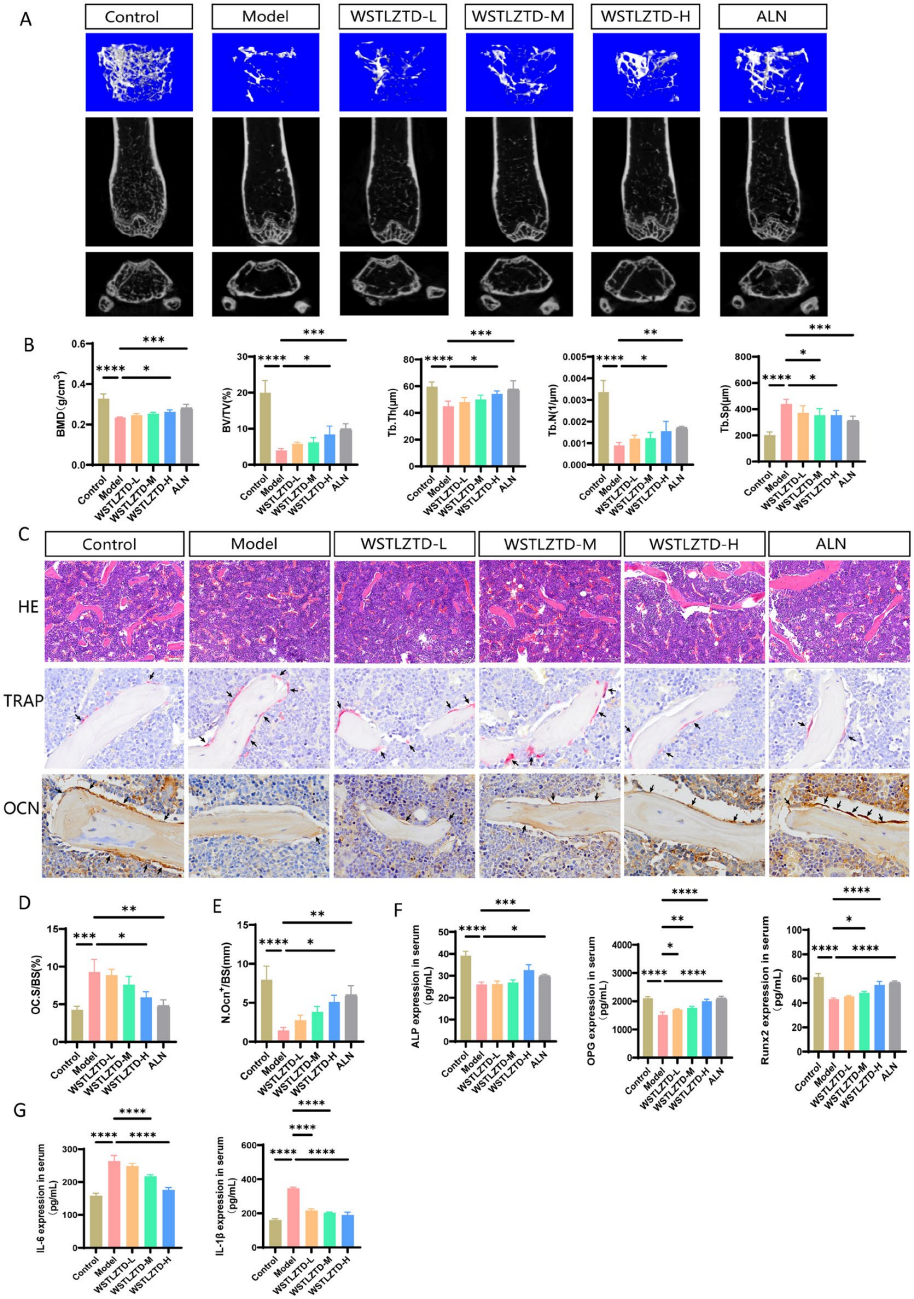
WSTLZTD ameliorates bone loss in SOP mice. (A) Representative three-dimensional micro-CT reconstructions of the distal femur in control, model and WSTLZTD-treated (low-, medium- and high-dose) groups. (B) Quantitative analysis of BMD, BV/TV, Tb.Th, Tb.N and Tb.Sp in the distal femur (*n* = 5). (C) Representative H&E-stained sections of the distal femur (*n* = 3; scale bar: 100 μm); representative images of TRAP staining and OCN of IHC assay in the distal femur (scale bar: 20 μm). The region highlighted in pink, indicated by the black arrow, denotes the presence of osteoclasts, whereas the region highlighted in brown, also indicated by the black arrow, denotes the presence of osteoblasts. (D) Quantitative analysis of Oc.S/BS (*n* = 3). (E) Quantitative analysis of OCN-positive cells per bone surface (N.OCN^+^/BS) in the distal femur (*n* = 3). (F) Serum levels of ALP, OPG and Runx2 across groups (*n* = 4). (G) Serum levels of inflammatory cytokines IL-6 and IL-1β across groups (*n* = 4). *p < 0.05, **p < 0.01, ***p < 0.001, ****p < 0.0001.

Aged mice exhibited SOP, characterized by marked bone mass reduction, trabecular microstructural deterioration, elevated bone fragility and decreased peak BMD (Wang ZX et al. 2024). Thus, 16-month-old mice were utilized as an SOP model, with 3-month-old mice serving as young controls. After 10 weeks of saline or WSTLZTD treatment, micro-CT analysis demonstrated that SOP model mice showed significant reductions in femoral BMD, BV/TV, Tb.N and Tb.Th, alongside increased Tb.Sp, compared to controls. Notably, WSTLZTD dose-dependently attenuated these pathological changes, restoring femoral trabecular microstructure (Figure 2(A,B)). H&E staining further confirmed that WSTLZTD increased femoral trabeculae (Figure 2(C)). The quantitative analysis for H&E staining is shown in Supplementary Data Figure S2(A). TRAP staining revealed elevated osteoclast surface per bone surface (Oc.S/BS) in model mice, which was suppressed by WSTLZTD (Figure 2(C,D)). Immunohistochemistry (IHC) showed reduced OCN-positive cells in model mice, an effect rescued by WSTLZTD (Figure 2(C,E)). Serum analysis demonstrated that WSTLZTD upregulated osteogenic markers (ALP, OPG, Runx2) (Figure 2(F)) and downregulated inflammatory cytokines (IL-6, IL-1β) (Figure 2(G)). Collectively, these data indicate that WSTLZTD mitigates bone loss and inflammatory dysregulation in SOP, with high-dose WSTLZTD yielding the most pronounced therapeutic benefits.

### WSTLZTD attenuates macrophage senescence in bone tissues and BMDMs of SOP mice

**Figure 3.**
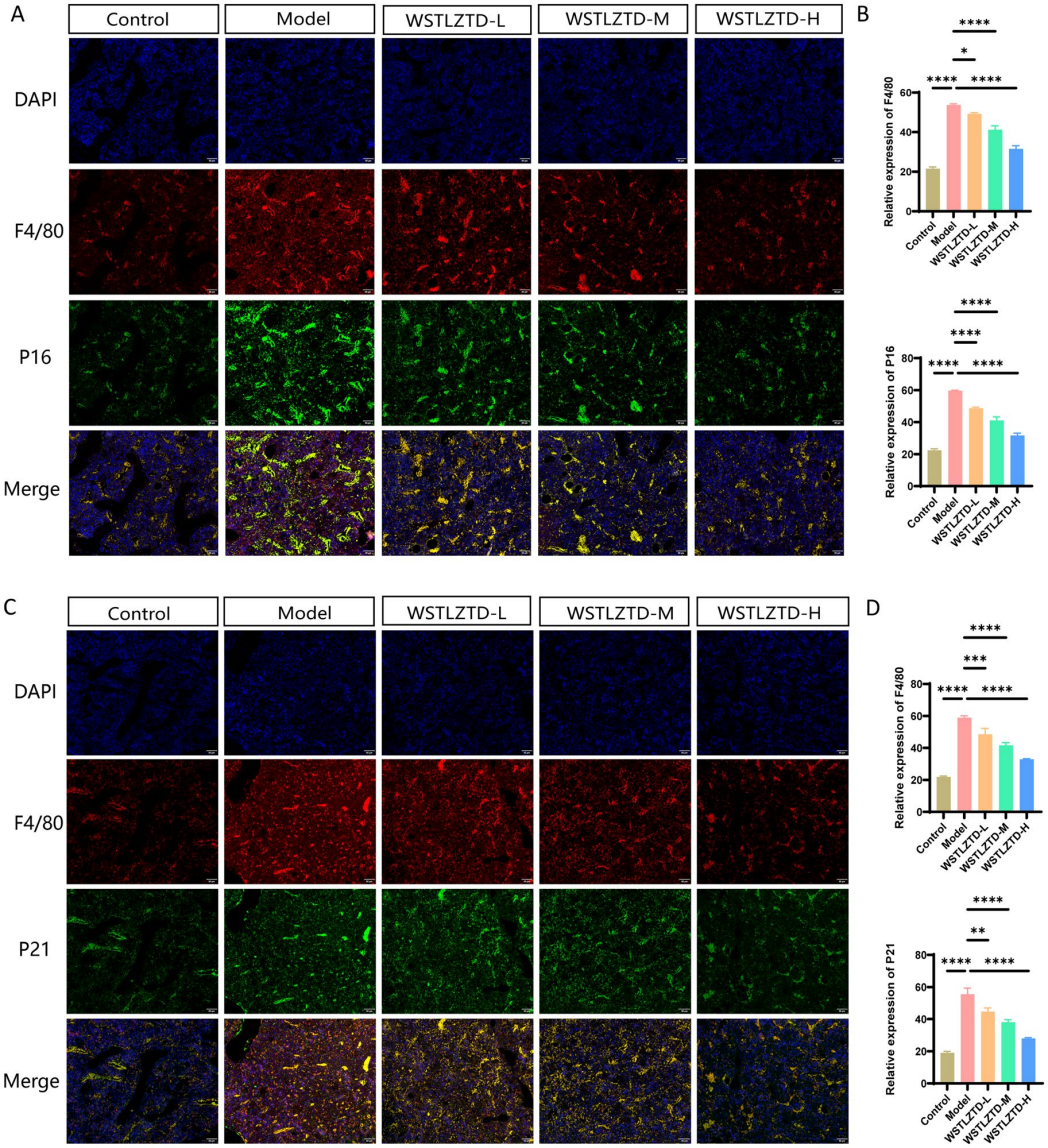
WSTLZTD attenuates macrophage senescence in the bone tissues of SOP mice. (A) Immunofluorescence staining of p16 expression in bone tissue macrophages across experimental groups (scale bar: 50 μm). (B) Quantitative analysis of the MFI of p16 in bone tissue macrophages (*n* = 3). (C) Immunofluorescence staining of p21 expression in bone tissue macrophages (scale bar: 50 μm). (D) Quantitative analysis of p21 MFI in bone tissue macrophages (*n* = 3). *p < 0.05, **p < 0.01, ***p < 0.001, ****p < 0.0001.

**Figure 4.**
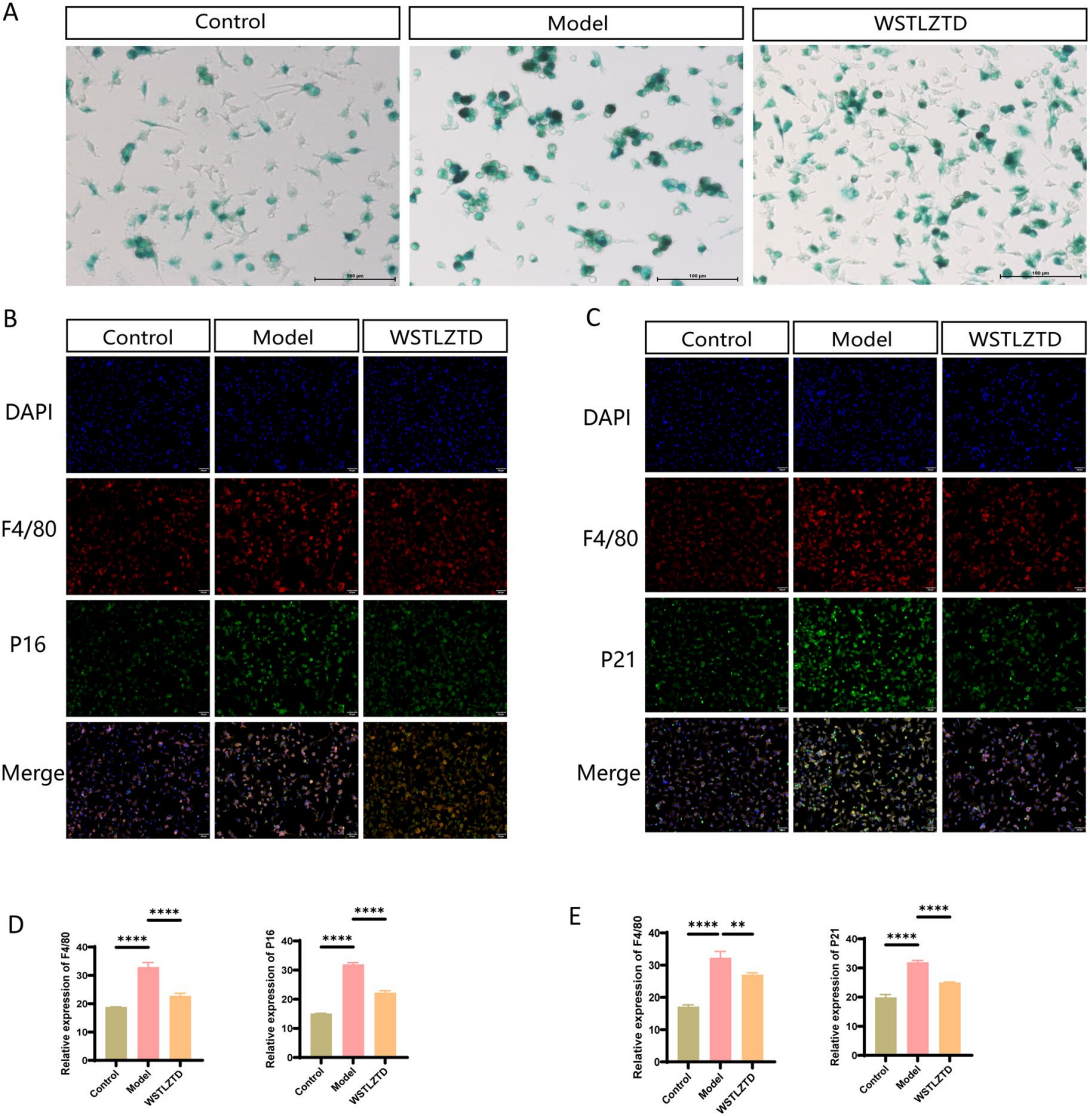
WSTLZTD alleviates BMDMs senescence in SOP mice. (A) β-gal activity staining of BMDMs from the control, model and WSTLZTD-treated groups to assess cellular senescence (*n* = 3; scale bar: 100 μm). (B,D) Representative immunofluorescence images of P16 expression in BMDMs (scale bar: 50 μm) and MFI quantitative analysis of F4/80 and P16 (*n* = 3). (C,E) Representative immunofluorescence images of P21 expression in BMDMs (scale bar: 50 μm) and quantitative analysis of F4/80 and P21 MFI (*n* = 3). *p < 0.05, **p < 0.01, ***p < 0.001, ****p < 0.0001.

F4/80 is extensively utilized as a macrophage surface marker (Cheng et al. 2024). Consistent with our earlier findings linking macrophage senescence to osteoporosis in d-galactose-induced models (Li M et al. 2024), immunofluorescence staining revealed decreased mean fluorescence intensity (MFI) of senescence markers (p16, p21) in femoral F4/80^+^ macrophages of SOP mice, which was dose-dependently restored by WSTLZTD (Figure 3(A–D)). β-Galactosidase (β-Gal) staining is a well-established method for detecting senescent cells, as it identifies high β-Gal activity commonly observed in senescent cells (Dimri et al. 1995; He et al. 2022; Cheng et al. 2024). To further validate the changes in cellular senescence, BMDMs isolated from control, model and high-dose WSTLZTD groups were subjected to β-Gal activity staining. Model mice exhibited a higher proportion of β-Gal positive senescent cells, whereas high-dose WSTLZTD significantly reduced senescent cell counts (Figure 4(A)). The quantitative analysis for β-Gal positive cells in BMDMs is shown in Supplementary Data Figure S2(B). Immunofluorescence confirmed that WSTLZTD downregulated p16 and p21 expression in BMDMs (Figure 4(B–E)), aligning with the trends observed in femoral macrophages.

### High-dose WSTLZTD-treated BMDMs enhance osteogenic differentiation and mitochondrial function in BMSCs

**Figure 5.**
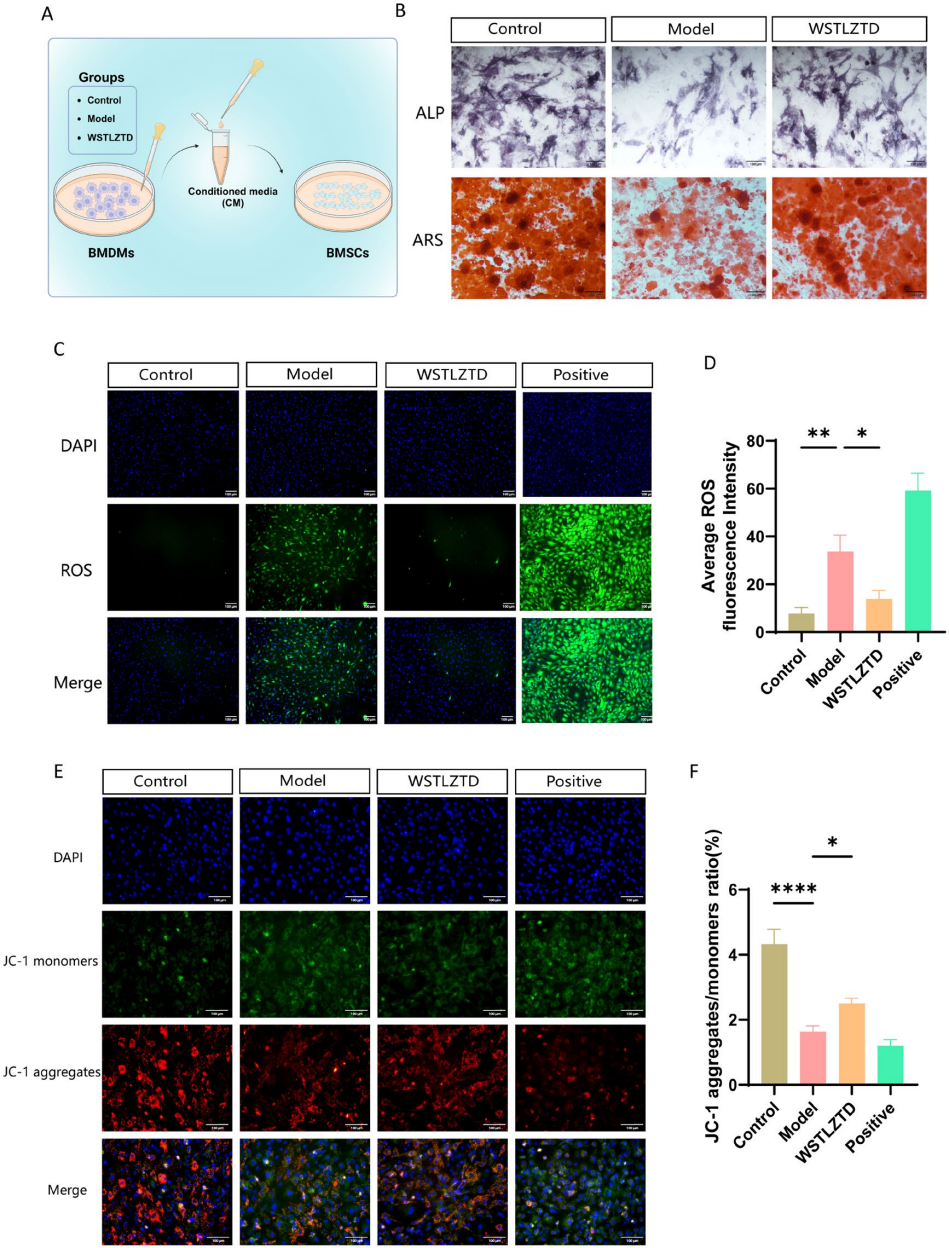
High-dose WSTLZTD suppress the effects of senescent BMDM-CM on BMSCs osteogenesis and mitochondrial metabolism. (A) CM from WSTLZTD-treated BMDMs was collected and applied to BMSCs. (B) Representative images of ALP and ARS staining in BMSCs treated with CM from control, model or high-dose WSTLZTD groups (*n* = 3; scale bar: 100 μm). (C,D) ROS levels in BMSCs treated with CM from each group were assessed by ROS staining (*n* = 3; scale bar: 100 μm). (E,F) ΔΨm in BMSCs treated with CM from each group was evaluated using JC-1 staining (*n* = 3; scale bar: 100 μm). *p < 0.05, **p < 0.01, ***p < 0.001, ****p < 0.0001.

To evaluate the effects of high-dose WSTLZTD-treated BMDMs on BMSC osteogenesis, BMDM-conditioned medium (CM) was co-cultured with BMSCs (Created in BioRender, Figure 5(A)). ALP and ARS staining revealed significantly increased mineralization in BMSCs treated with high-dose WSTLZTD-BMDM CM compared to the model group (Figure 5(B)). The quantitative analysis for ALP and ARS relative Integrated Density is shown in Supplementary Data Figure S2(C,D). Mitochondrial dysfunction, characterized by elevated ROS levels and reduced ΔΨm, was assessed (Gu et al. 2022; Videla et al. 2022). ROS staining demonstrated that BMSCs treated with high-dose WSTLZTD-BMDM CM exhibited lower ROS levels than those treated with model group (Figure 5(C,D)). JC-1 staining further confirmed enhanced ΔΨm (indicated by increased red fluorescence) in WSTLZTD-treated BMSCs (Figure 5(E,F)).

### Network pharmacology of WSTLZTD in the treatment of SOP

**Figure 6.**
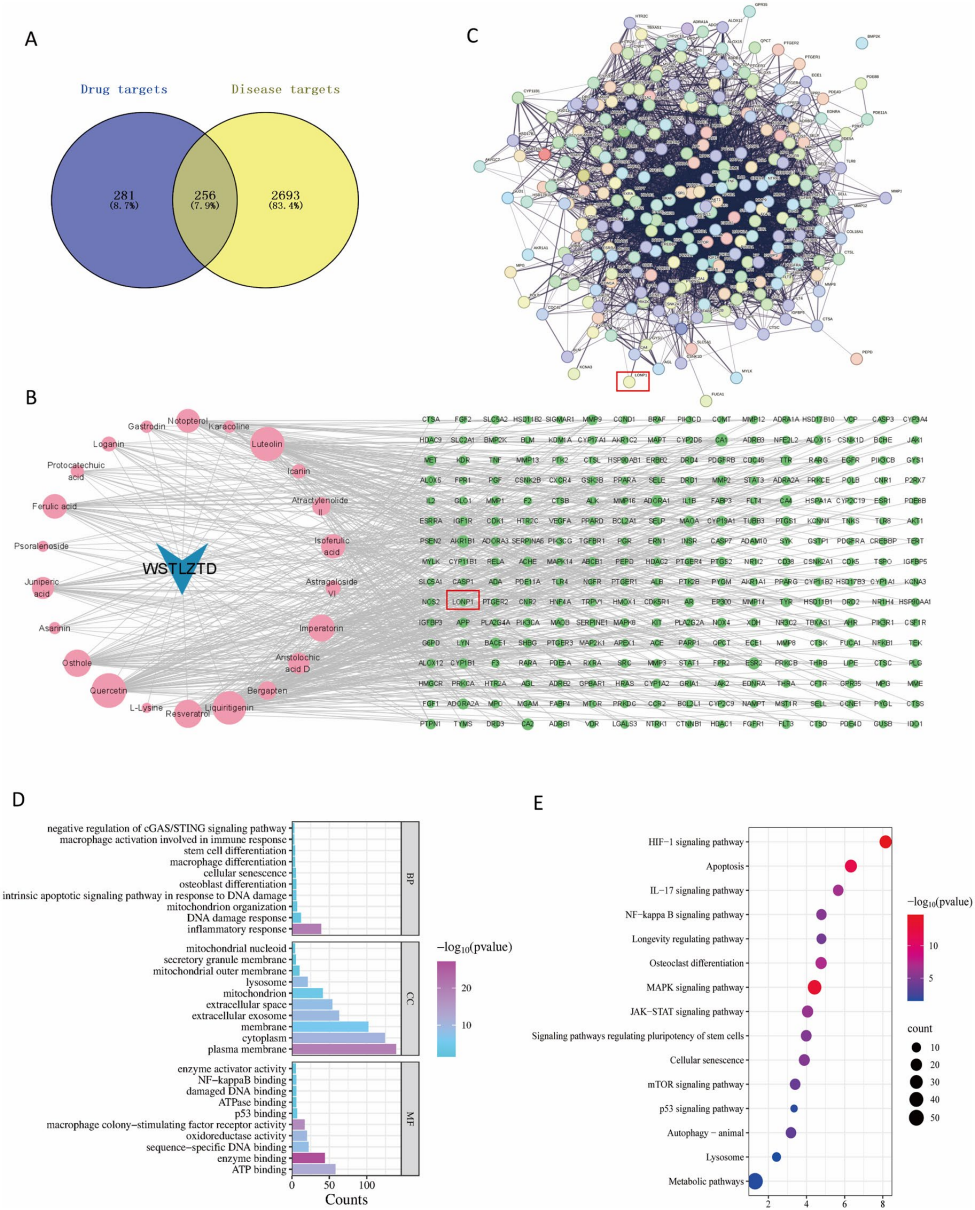
Network pharmacology of WSTLZTD in SOP therapy. (A) Venn diagram of drug targets and disease targets. (B) WSTLZTD active ingredient-target network. (C) PPI network of overlapping therapeutic targets. (D) GO enrichment analysis. (E) Pathway enrichment profiling *via* the KEGG database.

To explore the potential mechanisms of WSTLZTD against SOP, we conducted a network pharmacology analysis to identify key targets and signaling pathways. By interrogating the GeneCards and OMIM databases, we identified 2,949 SOP-associated targets. Intersection analysis between drug targets and disease targets revealed 256 overlapping targets (Figure 6(A)). Using Cytoscape 3.9.1, we constructed a bioactive compound-target network (Figure 6(B)), including key nodes such as NFKB1, LONP1, STAT3 and TLR8. These overlapping targets were further analyzed in the STRING database to generate a PPI network (Figure 6(C)). GO enrichment analysis demonstrated that biological processes were enriched in terms including negative regulation of the cGAS/STING signaling pathway, macrophage activation involved in immune response, cellular senescence, osteoblast differentiation and mitochondrial organization (Figure 6(D)). Cellular component terms featured mitochondrial outer membrane, lysosome and extracellular exosome, etc., while molecular functions included damaged DNA binding and ATPase activity (Figure 6(D)). KEGG pathway analysis revealed significant enrichment in longevity regulating pathway, cellular senescence, metabolic pathways and p53 signaling pathway (Figure 6(E)). Notably, the mitochondrial protease LONP1—essential for protein quality control and proteostasis during mitochondrial stress (Zanini et al. 2023)—has been widely reported to mitigate cellular senescence and senescence-associated pathologies through its ATP-dependent activity (Quirós et al. 2014; Bota and Davies 2016; Zhu et al. 2024). Furthermore, Activation of the cGAS/STING signaling pathway has been closely linked to mitochondrial energy metabolism dysfunction (Li MZ et al. 2022; Li L et al. 2024). Emerging study has revealed that secreted mitochondrial-derived vesicles exacerbate inflammatory responses in BMSCs via the cGAS/STING signaling axis, thereby impairing osteogenic differentiation (Pan et al. 2025). Intriguingly, our findings demonstrate that WSTLZTD alleviates macrophage senescence while enhancing mitochondrial energy metabolism and osteogenic differentiation in BMSCs. These observations suggest that WSTLZTD may regulate osteoblast differentiation by potentially modulating LONP1-mediated macrophage senescence.

### LONP1-targeted molecular docking analysis of key active compounds in WSTLZTD

**Figure 7.**
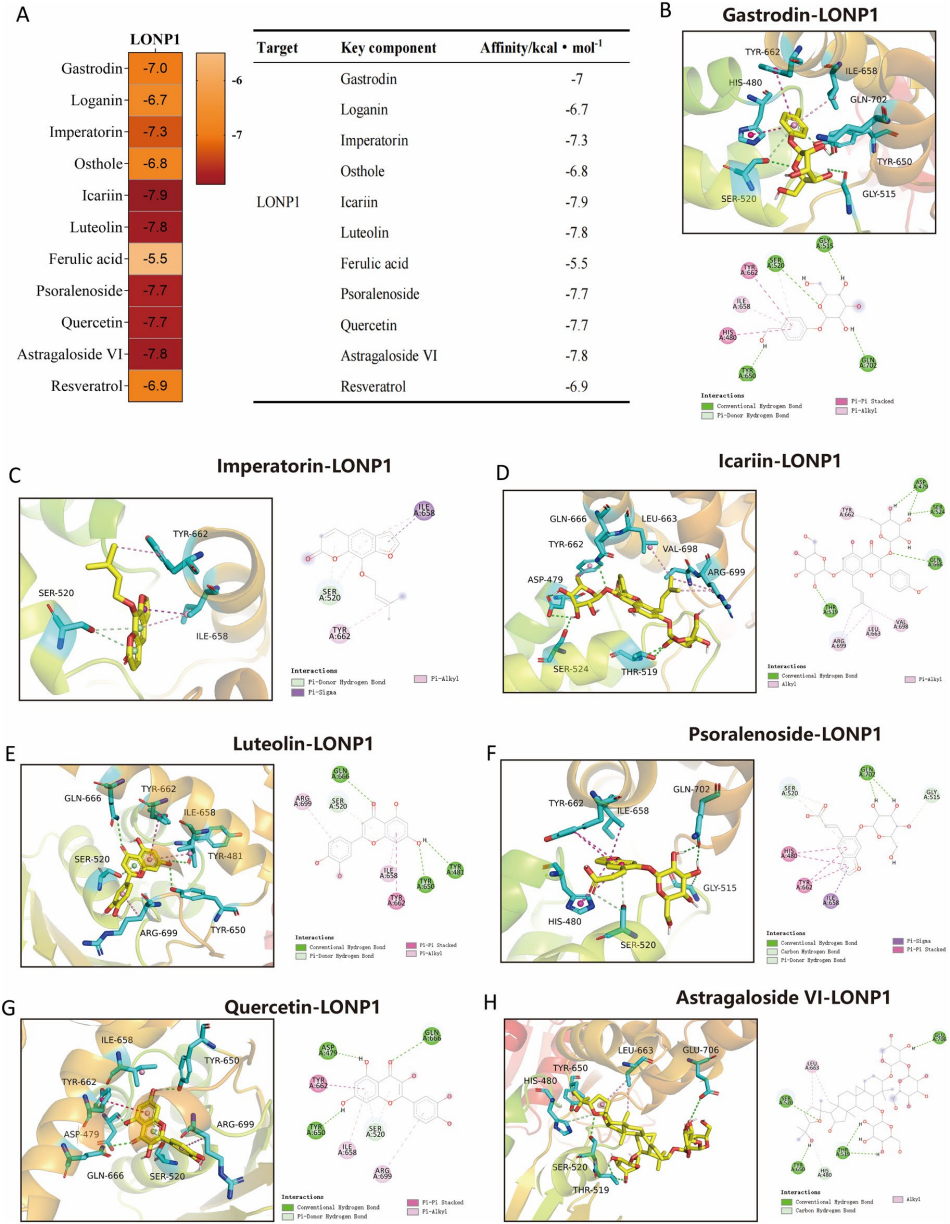
Molecular docking of key components in WSTLZTD with LONP1. (A) Binding energy table of key components with LONP1. (B-H) Molecular docking patterns between LONP1 and Gastrodin, Imperatorin, Icariin, Luteolin, Psoralenoside, Quercetin and Astragaloside VI. In the docking diagrams: yellow stick models represent active molecules; cyan stick structures indicate amino acid residues on the LONP1 protein; dashed lines denote chemical bonds between the active compounds and amino acid residues.

Eleven critical active compounds were further screened from the bioactive constituents of WSTLZTD, which may exert significant therapeutic effects in SOP. Molecular docking was performed between the target LONP1 and key 11 active compounds in WSTLZTD: Gastrodin, Loganin, Imperatorin, Osthole, Icariin, Luteolin, Ferulic acid, Psoralenoside, Quercetin, Astragaloside VI, Resveratrol. The binding energies of these compounds with LONP1 are presented in table (Figure 7(A)). Generally, docking scores with absolute values >4.25 indicate moderate activity, >5.0 suggest good binding affinity and >7.0 represent strong binding affinity. Complexes exhibiting binding energies < −7 kcal/mol were selected for graphical representation of molecular interactions (Figure 7(B–H)). The above results demonstrate favorable binding affinities between the key compounds in WSTLZTD and LONP1.

### High-dose WSTLZTD may Enhance BMSC osteogenic differentiation by alleviating BMDMs senescence via LONP1 and suppressing the cGAS/STING pathway in BMSCs

**Figure 8.**
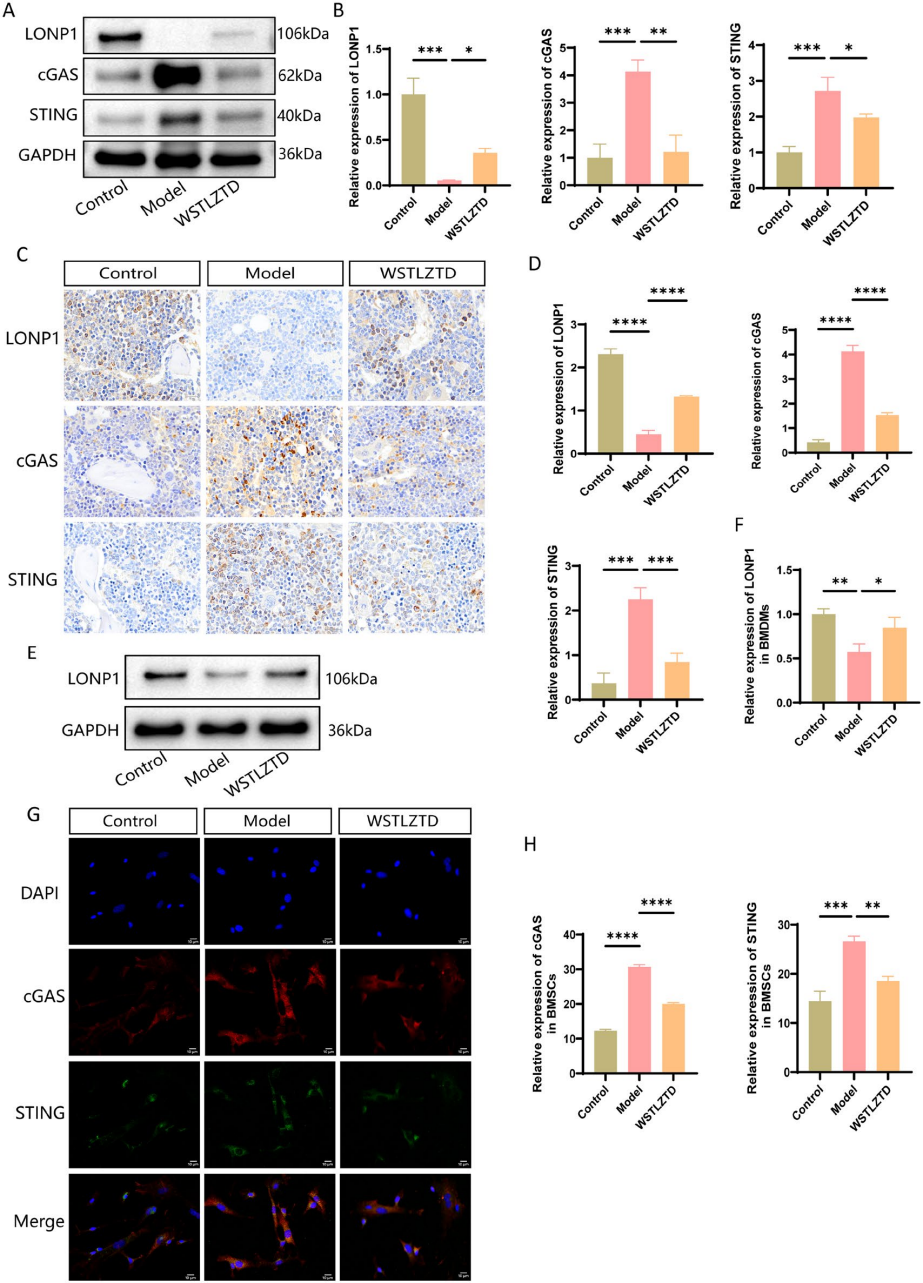
High-dose WSTLZTD may enhance BMSC osteogenic differentiation by alleviating BMDMs senescence via LONP1 and suppressing the cGAS/STING pathway in BMSCs. (A) WB analysis of LONP1, cGAS and STING protein levels in the femoral bone of mice. GAPDH was used as an internal control. (B) Quantitative analysis of LONP1, cGAS and STING protein expression using ImageJ software (*n* = 3). (C) Representative IHC images of LONP1, cGAS and STING in femoral bone sections (scale bar: 20 μm). (D) Quantitative analysis of LONP1, cGAS and STING protein expression from IHC (*n* = 3). (E) WB analysis of LONP1 protein levels in BMDMs across groups. GAPDH was used as an internal control. (F) Quantitative analysis of LONP1 expression in BMDMs using ImageJ software (*n* = 3). (G) Immunofluorescence staining of cGAS and STING in BMSCs treated with BMDM-CM (scale bar: 10 μm). (H) Quantitative analysis of cGAS and STING protein expression using ImageJ software (*n* = 3). *p < 0.05, **p < 0.01, ***p < 0.001, ****p < 0.0001.

To confirm the effects of high-dose WSTLZTD on LONP1 and cGAS/STING signaling, Western blot (WB) and IHC analyses were performed. As shown in Figure 8(A–D), LONP1 levels were significantly downregulated in the model group compared to controls, whereas cGAS and STING protein levels were markedly upregulated. High-dose WSTLZTD treatment attenuated these changes, upregulating LONP1 and suppressing cGAS/STING expression. Notably, high-dose WSTLZTD also elevated LONP1 expression in BMDMs (Figure 8(E,F)). Immunofluorescence further demonstrated that CM from high-dose WSTLZTD-treated BMDMs inhibited cGAS and STING expression in BMSCs (Figure 8(G,H)).

### WSTLZTD-containing serum promotes osteogenic differentiation of BMSCs via macrophage-derived CM in vitro

**Figure 9.**
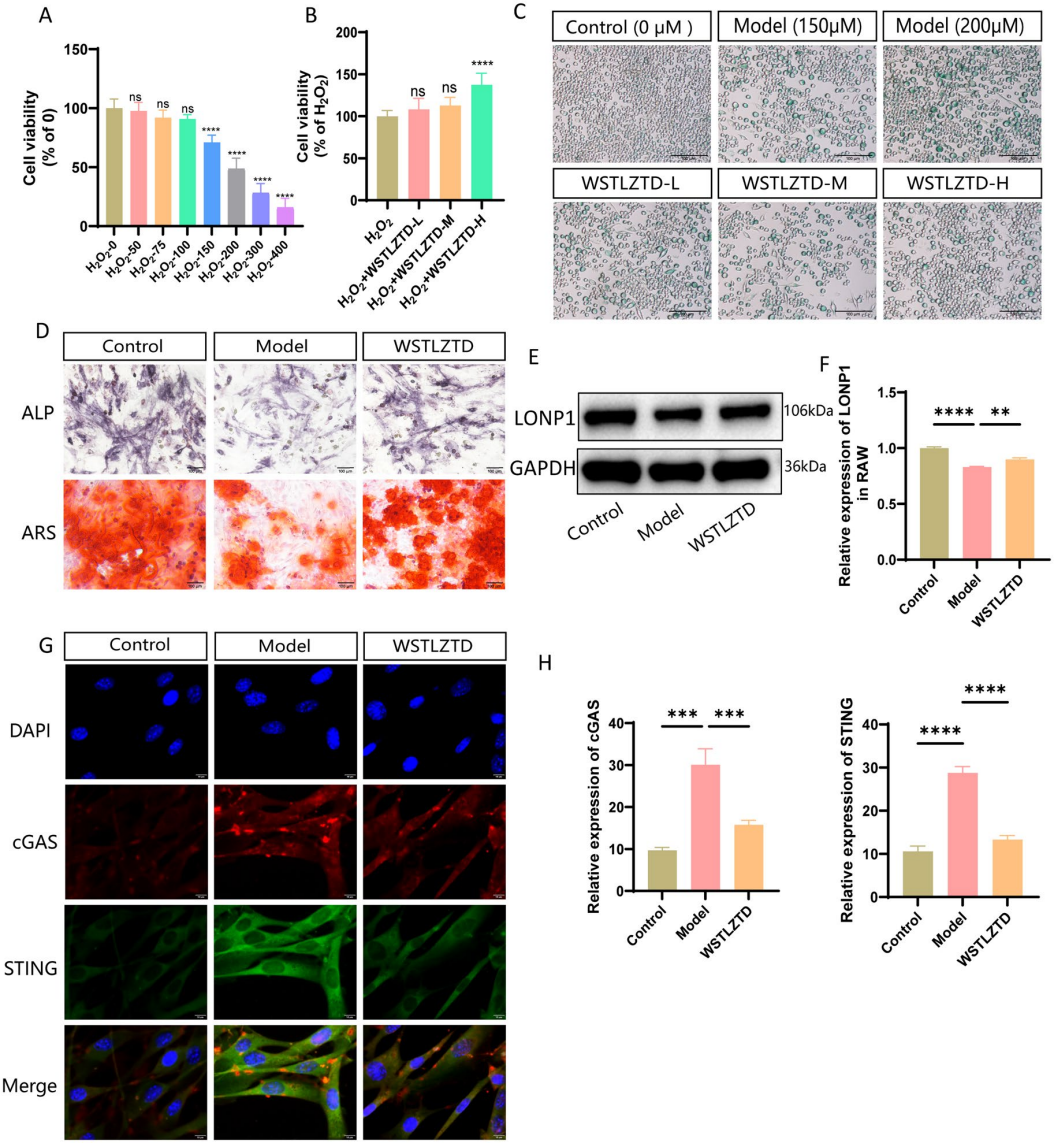
Effects of WSTLZTD-containing serum on senescent RAW264.7 macrophages. (A) Cell viability of RAW264.7 cells treated with different concentrations of H_2_O_2_ was assessed using the CCK-8 assay. (B) CCK-8 assay of senescent RAW264.7 cells treated with WSTLZTD-containing serum (low-, medium- and high-dose) (*n* = 3). (C) β-Gal activity staining to evaluate senescence in RAW264.7 cells from control, model and WSTLZTD-containing serum-treated groups (*n* = 3; scale bar: 100 μm). (D) Representative images of ALP and ARS staining in BMSCs treated with RAW-CM from control, model or high-dose WSTLZTD-containing serum groups (*n* = 3; scale bar: 100 μm). (E) WB analysis of LONP1 protein levels in RAW264.7 cells across groups. GAPDH was used as an internal control. (F) Quantitative analysis of LONP1 expression using ImageJ software (*n* = 3). (G) Immunofluorescence staining of cGAS and STING in BMSCs treated with RAW-CM from control, model or high-dose WSTLZTD-containing serum groups (scale bar: 10 μm). (H) Quantitative analysis of cGAS and STING expression using ImageJ software (*n* = 3). *p < 0.05, **p < 0.01, ***p < 0.001, ****p < 0.0001.

RAW264.7 cells, a murine monocyte-macrophage lineage (Thiyagarajan et al. 2023; Ikeda et al. 2024), were used to evaluate the effects of WSTLZTD-containing serum. H_2_O_2_ has been widely used to induce cellular senescence (Kim et al. 2022; Cheng et al. 2024). Additionally, our own preliminary research on macrophage senescence shows that H_2_O_2_-induced RAW264.7 cell senescence serves as a useful model to investigate key aspects of macrophage dysfunction, a critical driver of aging-related changes (Li M et al. 2024). In this study, CCK-8 assays revealed dose-dependent reductions in RAW264.7 cell viability with increasing H_2_O_2_ concentrations (Figure 9(A)). A concentration of 150 μM H_2_O_2_ (Merck, 88597) was selected to establish a senescence model. β-Gal staining showed that high-dose WSTLZTD-containing serum significantly attenuated senescence in H_2_O_2_-treated RAW264.7 cells compared to the model group (Figure 9(C)). The quantitative analysis for β-Gal positive cells in RAW264.7 cells is shown in Supplementary Data Figure S2(E). CCK-8 assays confirmed improved viability in senescent RAW264.7 cells treated with high-dose WSTLZTD serum (Figure 9(B)). ALP and ARS staining revealed enhanced osteogenic differentiation in BMSCs cultured with CM from WSTLZTD serum-treated RAW264.7 cells (Figure 9(D)). The quantitative analysis for ALP and ARS relative Integrated Density is shown in Supplementary Data Figure S2(F,G). Mechanistically, high-dose WSTLZTD serum upregulated LONP1 expression in senescent RAW264.7 cells (Figure 9(E,F)) and downregulated cGAS/STING levels in BMSCs (Figure 9(G,H)).

### WSTLZTD may mediate macrophage senescence potentially through LONP1 and then regulate BMSC osteogenesis via cGAS/STING pathway

**Figure 10.**
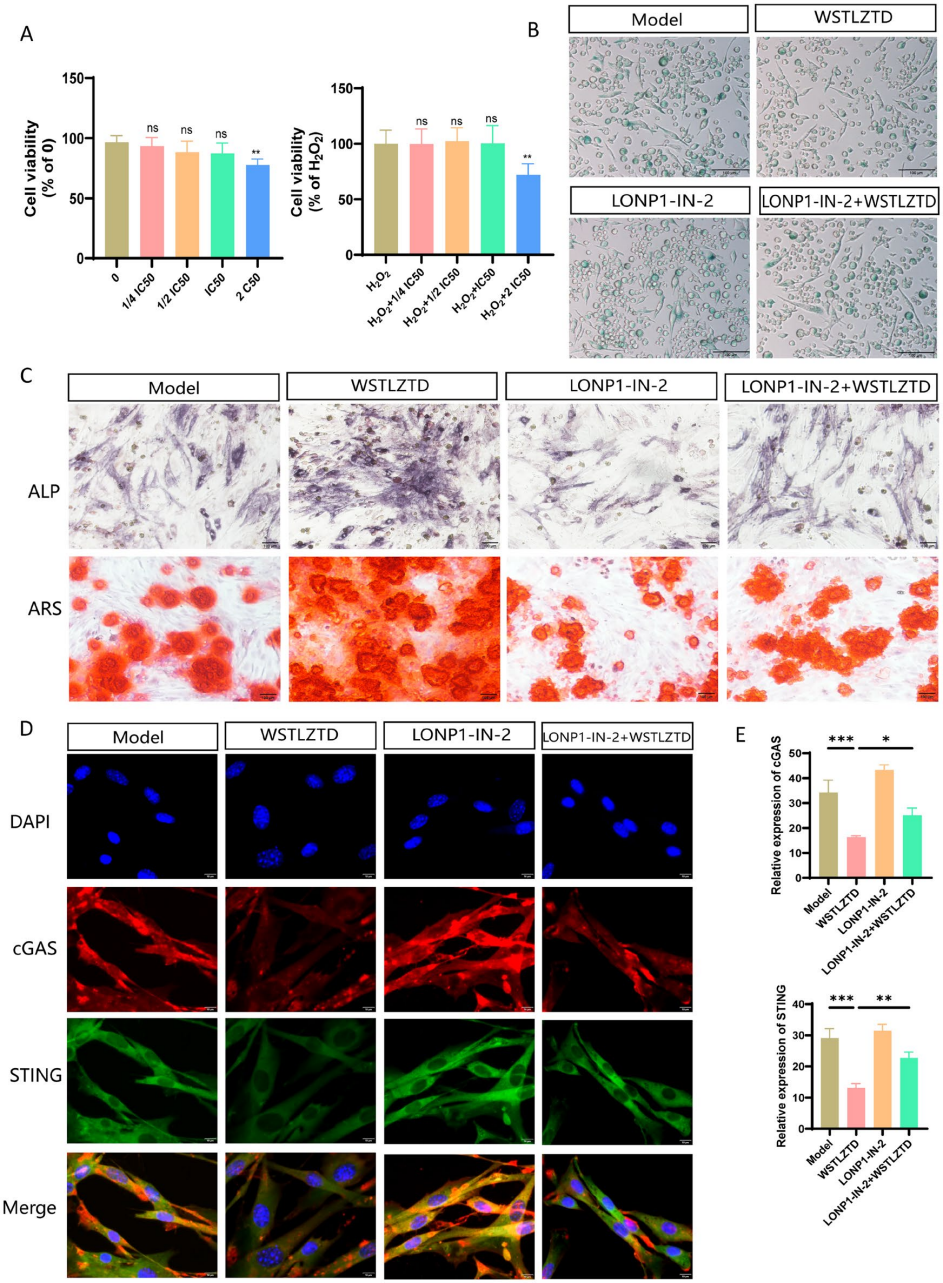
WSTLZTD may mediate macrophage senescence potentially through LONP1 and then regulate BMSC osteogenesis via cGAS/STING pathway. (A) The CCK-8 assay was employed to assess the viability of RAW264.7 cells and H_2_O_2_-induced senescent RAW264.7 cells, both subjected to treatment with the LONP1 inhibitor, LONP1-IN-2. (B) β-gal activity staining of RAW264.7 cells from the following groups: model, model + high-dose WSTLZTD-containing serum, model + vehicle serum + LONP1-IN-2 and model + high-dose WSTLZTD-containing serum + LONP1-IN-2 (*n* = 3; scale bar: 100 μm). (C) Representative images of ALP and ARS staining in BMSCs treated with RAW-CM from each group (*n* = 3; scale bar: 100 μm). (D) Immunofluorescence staining of cGAS and STING in BMSCs treated with RAW-CM from each group (scale bar: 10 μm). (E) Quantitative analysis of cGAS and STING protein expression using ImageJ software (*n* = 3). *p < 0.05, **p < 0.01, ***p < 0.001, ****p < 0.0001.

The selective LONP1 inhibitor LONP1-IN-2 (MCE, HY-153034) was utilized to validate the role of LONP1(Kingsley et al. 2021). To establish its therapeutic window, we first determined the maximum non-toxic dose of LONP1-IN-2, represented by its IC50 (half-maximal inhibitory concentration). Using CCK-8 assays, we identified the IC50 as the critical threshold for subsequent experiments, ensuring that LONP1-IN-2 treatments were pharmacologically effective while preserving cellular homeostasis (Figure 10(A)). Rescue experiments demonstrated that LONP1-IN-2 inhibited the anti-senescent effects of high-dose WSTLZTD in RAW264.7 cells, as evidenced by increased β-Gal positive cells (Figure 10(B)). Co-culture of BMSCs with CM from LONP1-IN-2-treated RAW264.7 cells attenuated the pro-osteogenic effects of WSTLZTD, as shown by reduced ALP and ARS staining (Figure 10(C)). The quantitative analysis for β-Gal positive cells in RAW264.7 cells, as well as the ALP and ARS relative Integrated Density are shown in Supplementary Data Figure S2(H–J). Concurrently, LONP1-IN-2 reactivated cGAS/STING signaling in BMSCs (Figure 10(D,E)).

## Discussion

With the global aging population, SOP has emerged as a widespread skeletal disorder, significantly increasing fracture risk and impairing quality of life. Unlike postmenopausal osteoporosis, which primarily affects women, SOP arises from age-related physiological decline and impacts both genders (Sözen et al. 2017). Current therapeutic options for osteoporosis are limited by efficacy, cost, side effects and patient adherence (Curraj and Gonzalez Rodriguez 2023). TCM, particularly herbal formulations, offers unique advantages in the treatment of osteoporosis (Su et al. 2024). WSTLZTD, a plant-based TCM formula, has demonstrated anti-osteoporotic potential in prior studies using ovariectomized (OVX), d-galactose-induced or SAMP6 murine models (Wang L et al. 2023; Li M et al. 2024, 2025). However, its efficacy in naturally aged SOP models remained unvalidated.

In this study, we validated WSTLZTD’s therapeutic effects in a natural aging SOP mouse model. Micro-CT and H&E staining revealed increases in Tb.N and thickness with WSTLZTD treatment. OCN, a marker of bone formation (Shen et al. 2022), and TRAP, an indicator of osteoclast activity (Magnusson and Ransjö 2024), were synergistically analyzed to assess bone remodeling. WSTLZTD elevated OCN^+^ cell counts while suppressing TRAP^+^ osteoclast surface area, indicating enhanced osteoblast activity and attenuated bone resorption. These findings align with WSTLZTD’s dual role in promoting osteogenesis and inhibiting osteoclastogenesis, rebalancing bone remodeling in SOP. Serum analysis further corroborated these results, showing upregulated osteogenic biomarkers (ALP, OPG, Runx2) (Ishihata et al. 2022; Knani et al. 2025) and reduced pro-inflammatory cytokines such as IL-6 and IL-1β (Liu et al. 2023; Chen Z et al. 2025), which are elevated in chronic inflammatory bone loss.

Macrophages, as one of the most active innate immune cells in bone tissue, play a pivotal role in maintaining normal bone metabolic homeostasis (Hu et al. 2023). Recent studies have increasingly focused on macrophage senescence and its pathological contributions to SOP. For instance, Cheng et al. (2024) demonstrated that lipoteichoic acid (LTA) treatment alleviates bone marrow macrophage senescence and improves SOP by suppressing mTOR phosphorylation via the β-catenin/FOXO1/REDD1 pathway. They also observed altered mRNA expression of senescence-associated genes, such as p16, p21 and p53, in LTA-treated senescent macrophages. In our study, we found that WSTLZTD significantly reduced the MFI of senescence markers p16 and p21 in femoral F4/80^+^ macrophages compared to the untreated model group. This reduction was dose-dependent, further supporting the superior anti-senescence efficacy of high-dose WSTLZTD. To elucidate the specific mechanism, BMDMs were isolated from different treatment groups. β-Gal staining revealed a higher proportion of β-Gal positive senescent cells in the model group compared to young controls, whereas high-dose WSTLZTD markedly reduced senescent cell counts. Immunofluorescence confirmed that high-dose WSTLZTD effectively downregulated p16 and p21 expression in BMDMs, consistent with the senescence attenuation observed in femoral macrophages. Interestingly, although F4/80 is a common marker for macrophages, it may also label monocytes in certain situations (Tosun et al. 2022). Therefore, further experiments could help more precisely differentiate and determine the proportion of macrophages.

A growing body of evidence highlights the pivotal role of osteoimmune microenvironment imbalance in the pathogenesis of SOP (Yang and Liu 2021; Huang F et al. 2022). While earlier studies predominantly focused on the impact of macrophages on osteoblast and osteoclast differentiation, recent advances reveal that macrophage functional states critically interact with BMSCs within the bone marrow niche, dictating their differentiation fate (Chen L et al. 2021; Wang D et al. 2023). In this study, we further explored the regulatory potential of high-dose WSTLZTD on BMSC osteogenic differentiation. BMSCs were co-cultured with CM from BMDMs of different treatment groups. Results demonstrated that CM from high-dose WSTLZTD-treated BMDMs significantly enhanced BMSC osteogenic differentiation compared to the model group, as evidenced by elevated ALP activity and intensified ARS staining. Mitochondrial dysfunction, characterized by elevated ROS levels and diminished ΔΨm, is implicated in osteoporosis and other degenerative disorders (Cai et al. 2023). We further assessed mitochondrial function in BMSCs treated with BMDM-CM using ROS and JC-1 staining. BMSCs exposed to high-dose WSTLZTD-BMDM-CM exhibited significantly reduced ROS levels and restored ΔΨm. These findings suggest that WSTLZTD may ameliorate the osteoimmune microenvironment by modulating BMDMs senescence, thereby promoting BMSC osteogenesis. However, the precise molecular mechanisms underlying WSTLZTD’s regulation of bone metabolism, including its key targets and signaling pathways, require further investigation.

Recent research has reported that LONP1 expression is downregulated in age-related skeletal disorders, and its knockout significantly elevates ROS and mitochondrial peroxide levels, leading to loss of ΔΨm (He et al. 2022). Similarly, Venkatesh et al. demonstrated that LONP1 knockdown increases mitochondrial superoxide production, causing oxidative damage to mitochondrial proteins (Venkatesh et al. 2019). These findings suggest that LONP1 deficiency may exacerbate aging-related pathologies. The cytosolic DNA sensor cGAS can bind to oxidized DNA and activate the STING pathway, promoting secretion of senescence-associated factors and suppressing mitochondrial energy metabolism, thereby accelerating aging-related disease progression (Jiang et al. 2023; Fan et al. 2024). The latest studies have also found that intercellular communication mediators can influence the osteogenic differentiation of BMSCs through the cGAS-STING pathway (Pan et al. 2025). Huang Y et al. (2025) demonstrated that inhibiting the cGAS-STING pathway activation induced by oxidative stress effectively restored the metabolism and osteogenic differentiation of aging BMSCs. Therefore, these findings provide intriguing clues for understanding the potential relationship between LONP1 and the cGAS/STING pathway. Additionally, Network pharmacology analysis of WSTLZTD in the treatment of SOP suggests that WSTLZTD-mediated macrophage senescence may be associated with the target protein LONP1. The 11 active ingredients identified through LC–MS/MS analysis were shown to have strong binding affinity with LONP1 according to molecular docking. For instance, Resveratrol has a binding energy of −6.9 kcal/mol with LONP1, and the remaining seven compounds (Gastrodin, Imperatorin, Icariin, Luteolin, Psoralenoside, Quercetin and Astragaloside VI) exhibited binding energies of −7, −7.3, −7.9, −7.8, −7.7, −7.7 and −7.8 kcal/mol, all of which were stronger than the binding energy between Resveratrol and LONP1. However, there have been reports confirming that Resveratrol can regulate LONP1 or potentially exert mitochondrial functional effects through LONP1-mediated mechanisms in cells (Raghubeer et al. 2015; Kalvala et al. 2020; He et al. 2022). This suggests a high probability that the other active ingredients in WSTLZTD bind to and regulate LONP1. Moreover, WB and IHC analyses in our study revealed marked downregulation of LONP1 in bone tissue from the model group compared to controls, which was upregulated by WSTLZTD intervention. Furthermore, the expression trends of LONP1 in BMDMs mirrored those observed in bone tissue, strongly supporting the hypothesis that WSTLZTD may regulate LONP1 in senescent macrophages. GO and KEGG analyses indicated that negative regulation of the cGAS/STING signaling pathway may influence osteoblast differentiation. To investigate whether WSTLZTD modulates BMSC osteogenesis through macrophage LONP1-dependent regulation of cGAS/STING, we analyzed their levers in bone tissue and BMSCs. WSTLZTD downregulated cGAS and STING expression in bone tissue. Notably, CM from WSTLZTD-treated BMDMs suppressed cGAS/STING pathway activation in BMSCs.

To further validate the therapeutic potential of WSTLZTD-containing serum in modulating macrophage senescence and promoting BMSC osteogenesis, we employed the LONP1-specific inhibitor LONP1-IN-2. A senescence model was established using H_2_O_2_-treated RAW264.7 murine macrophages. WSTLZTD-containing serum significantly reduced β-Gal positive senescent cells and enhanced cell viability via CCK-8 assays, indicating its efficacy in alleviating macrophage senescence. CM from WSTLZTD-treated macrophages was then applied to BMSCs, which exhibited enhanced osteogenic differentiation, as evidenced by elevated ALP activity and ARS staining intensity compared to the model group. Notably, LONP1-IN-2 was found to diminish WSTLZTD’s anti-senescent and pro-osteogenic effects. Specifically, CM from LONP1-IN-2-treated macrophages failed to suppress cGAS/STING pathway activation in BMSCs, attenuating the osteogenic enhancement observed in the WSTLZTD group. These findings collectively demonstrate that WSTLZTD regulates BMSC osteogenesis potentially through LONP1-mediated attenuation of macrophage senescence and subsequent inhibition of cGAS/STING signaling.

## Conclusions

**Figure 11.**
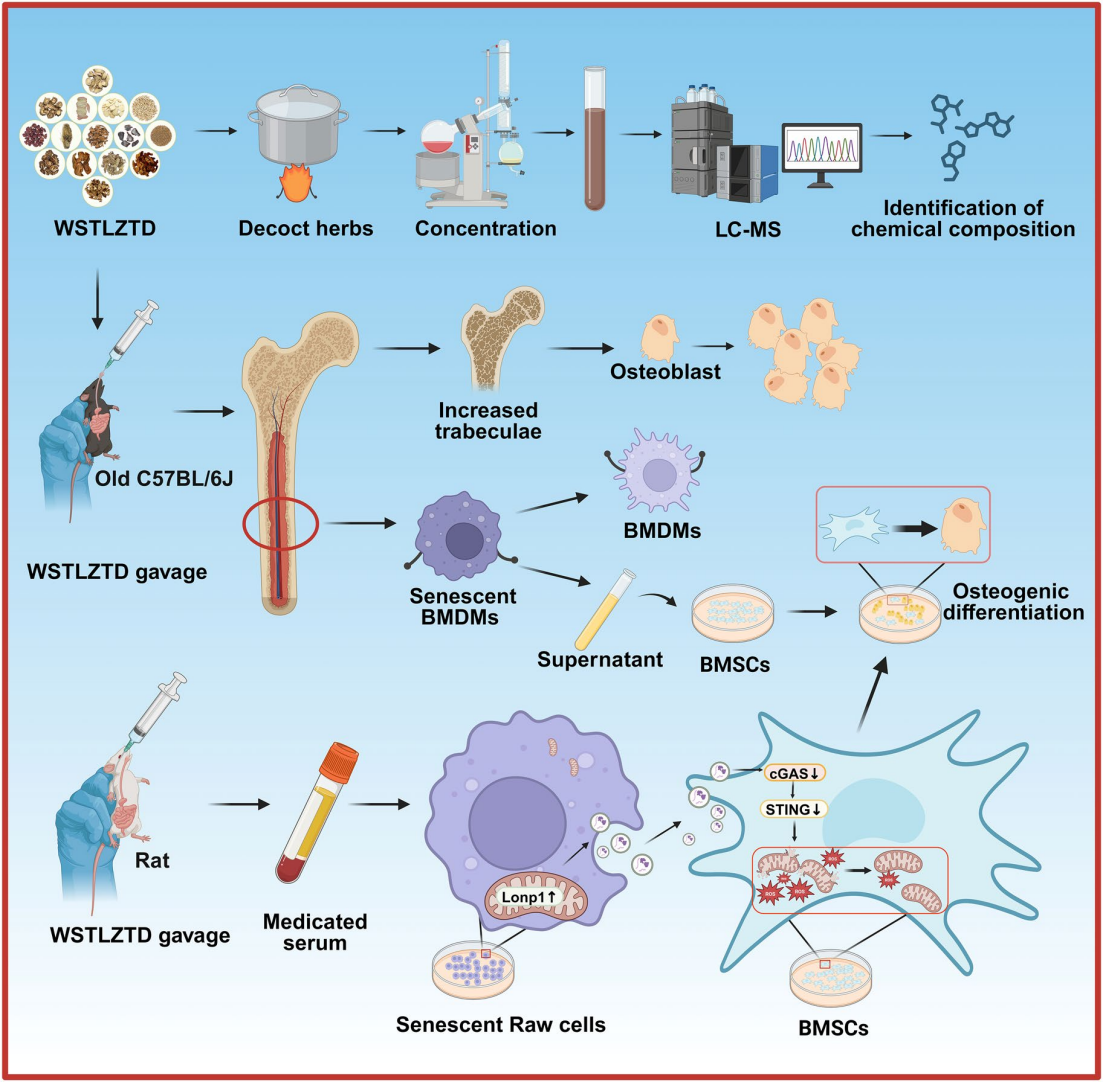
Schematic illustration of the mechanism underlying WSTLZTD-mediated protection against SOP. The diagram summarizes how WSTLZTD attenuates macrophage senescence, consequently suppressing cGAS/STING pathway activation in BMSCs to promote osteogenic differentiation and mitigate bone loss in SOP.

In summary, this study reveals that WSTLZTD ameliorates osteoporosis by regulating macrophage senescence via LONP1, thereby suppressing cGAS/STING pathway activation in BMSCs to enhance osteogenic differentiation (Created in BioRender, Figure 11). These findings highlight WSTLZTD’s potential in aging-related bone disorders, particularly through immune-bone metabolic modulation. However, this study has several limitations that need to be considered. (1) The clinical limitations of WSTLZTD are primarily attributed to the insufficient evidence derived from large-scale, evidence-based clinical studies concerning SOP. (2) WSTLZTD bioavailability, pharmacokinetics or potential herb-drug interactions have not been fully explored or validated and will require further detailed investigation in the future. (3) Direct interaction of bioactive components of WSTLZTD with LONP1, including binding and regulatory abilities, requires further validation through SPR experiments and more detailed studies. (4) Stricter experimental validation of molecular mechanisms, including additional genetic validation (e.g., LONP1 siRNA) *in vitro* and LONP1 knockout *in vivo*, should take into account the potential lethality associated with the latter (Quirós et al. 2014). Future studies could attempt to validate this by using macrophage-specific conditional LONP1 knockout models, such as the LONP1fl/fl;Lyz2-Cre+ (cKO) mouse model, which may help achieve LONP1 knockout in BMDMs. These above will provide an interesting avenue for further research.

## Supplementary Material

Figure S2.docx

Figure S1.docx

## Data Availability

The datasets used and analyzed during the current study are available from the corresponding author on reasonable request.

## References

[CIT0001] Arron JR, Choi Y. 2000. Bone versus immune system. Nature. 408(6812):535–536. doi:10.1038/35046196. 11117729

[CIT0002] Bai L, Liu Y, Zhang X, Chen P, Hang R, Xiao Y, Wang J, Liu C. 2023. Osteoporosis remission via an anti-inflammaging effect by icariin activated autophagy. Biomaterials. 297:122125. doi:10.1016/j.biomaterials.2023.122125. 37058900

[CIT0003] Bota DA, Davies KJ. 2016. Mitochondrial Lon protease in human disease and aging: including an etiologic classification of Lon-related diseases and disorders. Free Radic Biol Med. 100:188–198. doi:10.1016/j.freeradbiomed.2016.06.031. 27387767 PMC5183306

[CIT0004] Cai W, Zhang J, Yu Y, Ni Y, Wei Y, Cheng Y, Han L, Xiao L, Ma X, Wei H, et al. 2023. Mitochondrial transfer regulates cell fate through metabolic remodeling in osteoporosis. Adv Sci. 10(4):e2204871. doi:10.1002/advs.202204871. PMC9896036 36507570

[CIT0005] Chen L, Qiao P, Liu H, Shao L. 2021. Amorphous calcium phosphate NPs mediate the macrophage response and modulate BMSC osteogenesis. Inflammation. 44(1):278–296. doi:10.1007/s10753-020-01331-9. 32939669

[CIT0006] Chen Z, Yang G, Su W, He S, Wang Y. 2025. Serum IL-6 and TGF-β1 concentrations as diagnostic biomarkers in elderly male patients with osteoporosis. Eur Spine J. 34(2):513–521. doi:10.1007/s00586-024-08553-7. 39570334

[CIT0007] Cheng W, Fu Y, Lin Z, Huang M, Chen Y, Hu Y, Lin Q, Yu B, Liu G. 2024. Lipoteichoic acid restrains macrophage senescence via β-catenin/FOXO1/REDD1 pathway in age-related osteoporosis. Aging Cell. 23(3):e14072. doi:10.1111/acel.14072. 38126583 PMC10928565

[CIT0008] Curraj E, Gonzalez Rodriguez E. 2023. Side effects of osteoporosis treatments: how to explain them to patients? Rev Med Suisse. 19(823):746–751. 37133954 10.53738/REVMED.2023.19.823.746

[CIT0009] Dimri GP, Lee X, Basile G, Acosta M, Scott G, Roskelley C, Medrano EE, Linskens M, Rubelj I, Pereira-Smith O. 1995. A biomarker that identifies senescent human cells in culture and in aging skin in vivo. Proc Natl Acad Sci U S A. 92(20):9363–9367. doi:10.1073/pnas.92.20.9363. 7568133 PMC40985

[CIT0010] Fan K, Dong N, Fang M, Xiang Z, Zheng L, Wang M, Shi Y, Tan G, Li C, Xue Y. 2024. Ozone exposure affects corneal epithelial fate by promoting mtDNA leakage and cGAS/STING activation. J Hazard Mater. 465:133219. doi:10.1016/j.jhazmat.2023.133219. 38101018

[CIT0011] Gu MJ, Hyon JY, Lee HW, Han EH, Kim Y, Cha YS, Ha SK. 2022. Glycolaldehyde, an advanced glycation end products precursor, induces apoptosis via ROS-mediated mitochondrial dysfunction in renal mesangial cells. Antioxidants. 11(5):934. doi:10.3390/antiox11050934. PMC9137959 35624799

[CIT0012] He Y, Ding Q, Chen W, Lin C, Ge L, Ying C, Xu K, Wu Z, Xu L, Ran J, et al. 2022. LONP1 downregulation with ageing contributes to osteoarthritis via mitochondrial dysfunction. Free Radic Biol Med. 191:176–190. doi:10.1016/j.freeradbiomed.2022.08.038. 36064070

[CIT0013] Hu K, Shang Z, Yang X, Zhang Y, Cao L. 2023. Macrophage polarization and the regulation of bone immunity in bone homeostasis. J Inflamm Res. 16:3563–3580. doi:10.2147/JIR.S423819. 37636272 PMC10460180

[CIT0014] Huang F, Wong P, Li J, Lv Z, Xu L, Zhu G, He M, Luo Y. 2022. Osteoimmunology: the correlation between osteoclasts and the Th17/Treg balance in osteoporosis. J Cell Mol Med. 26(13):3591–3597. doi:10.1111/jcmm.17399. 35633138 PMC9258696

[CIT0015] Huang Y, Mao J, Li Z, Wang W, Ni Z, Cai F, Tang J, Wang W, Zhang L, Zhou L, et al. 2025. Signal converter-based therapy platform promoting aging bone healing by improving permeability of the mitochondrial membrane. Adv Mater. 37(27):e2500156. doi:10.1002/adma.202500156. 40289881

[CIT0016] Ikeda R, Kimura C, Nihashi Y, Umezawa K, Shimosato T, Takaya T. 2024. Osteogenic CpG oligodeoxynucleotide, iSN40, inhibits osteoclastogenesis in a TLR9-dependent manner. Life. 14(12):1572. 10.3390/life14121572 PMC11679607 39768281

[CIT0017] Ishihata K, Seong CH, Kibe T, Nakazono K, Mardiyantoro F, Tada R, Nishimura M, Matsuguchi T, Nakamura N. 2022. Lipoteichoic acid and lipopolysaccharides are affected by p38 and inflammatory markers and modulate their promoting and inhibitory effects on osteogenic differentiation. Int J Mol Sci. 23(20):12633. doi:10.3390/ijms232012633. 36293485 PMC9604490

[CIT0018] Jiang A, Liu J, Wang Y, Zhang C. 2023. cGAS-STING signaling pathway promotes hypoxia-induced renal fibrosis by regulating PFKFB3-mediated glycolysis. Free Radic Biol Med. 208:516–529. doi:10.1016/j.freeradbiomed.2023.09.011. 37714438

[CIT0019] Kalvala AK, Yerra VG, Sherkhane B, Gundu C, Arruri V, Kumar R, Kumar A. 2020. Chronic hyperglycemia impairs mitochondrial unfolded protein response and precipitates proteotoxicity in experimental diabetic neuropathy: focus on LonP1 mediated mitochondrial regulation. Pharmacol Rep. 72(6):1627–1644. doi:10.1007/s43440-020-00147-6. 32720218

[CIT0020] Kim HJ, Kim WJ, Shin HR, Yoon HI, Moon JI, Lee E, Lim JM, Cho YD, Lee MH, Kim HG, et al. 2022. ROS-induced PADI2 downregulation accelerates cellular senescence via the stimulation of SASP production and NFκB activation. Cell Mol Life Sci. 79(3):155. doi:10.1007/s00018-022-04186-5. 35218410 PMC8882118

[CIT0021] Kingsley LJ, He X, McNeill M, Nelson J, Nikulin V, Ma Z, Lu W, Zhou VW, Manuia M, Kreusch A, et al. 2021. Structure-based design of selective LONP1 inhibitors for probing in vitro biology. J Med Chem. 64(8):4857–4869. doi:10.1021/acs.jmedchem.0c02152. 33821636

[CIT0022] Knani L, Venditti M, Rouis H, Minucci S, Messaoudi I. 2025. Effects of dopaminergic neuron degeneration on osteocyte apoptosis and osteogenic markers in 6-OHDA male rat model of Parkinson’s disease. Bone. 190:117271. doi:10.1016/j.bone.2024.117271. 39369834

[CIT0023] Li CJ, Xiao Y, Sun YC, He WZ, Liu L, Huang M, He C, Huang M, Chen KX, Hou J, et al. 2021. Senescent immune cells release grancalcin to promote skeletal aging. Cell Metab. 33(10):1957–1973.e6. e1956. doi:10.1016/j.cmet.2021.08.009. 34614408

[CIT0024] Li L, Liu F, Feng C, Chen Z, Zhang N, Mao J. 2024. Role of mitochondrial dysfunction in kidney disease: insights from the cGAS-STING signaling pathway. Chin Med J. 137(9):1044–1053. doi:10.1097/CM9.0000000000003022. 38445370 PMC11062705

[CIT0025] Li M, Niu Y, Tian L, Zhang T, Zhou S, Wang L, Sun J, Wumiti T, Chen Z, Zhou Q, et al. 2024. Astragaloside IV alleviates macrophage senescence and d-galactose-induced bone loss in mice through STING/NF-κB pathway. Int Immunopharmacol. 129:111588. doi:10.1016/j.intimp.2024.111588. 38290207

[CIT0026] Li M, Niu Y, Zhang T, Yang H, Tian L, Zhou S, Wumiti T, Sun J, Zhou Q, Zuo X, et al. 2025. Wen-Shen-Tong-Luo-Zhi-Tong-Decoction inhibits bone loss in senile osteoporosis model mice by promoting testosterone production. J Ethnopharmacol. 338(Pt 2):119033. doi:10.1016/j.jep.2024.119033. 39515680

[CIT0027] Li MZ, Wen XY, Liu XQ, Wang YQ, Yan L. 2022. LPS-induced activation of the cGAS-STING pathway is regulated by mitochondrial dysfunction and mitochondrial DNA leakage in endometritis. J Inflamm Res. 15:5707–5720. doi:10.2147/JIR.S374318. 36238763 PMC9550576

[CIT0028] Liu S, Li J, Zhang M. 2023. Determination of immune factor levels in serum and local hematoma samples of osteoporotic fracture patients and clinical study of the effect of active vitamin D3 treatment on immune factor levels. J Orthop Surg Res. 18(1):291. doi:10.1186/s13018-023-03777-7. 37038178 PMC10088267

[CIT0029] Ma Y, Wang Z, Wang P. 2010. Clinical study on treating 45 cases of osteoporosis with self-prepared Wenshen Tongluo Zhitong prescription. J Trad Chin Med. 38(01):103–104.

[CIT0030] Magnusson C, Ransjö M. 2024. Orthosilicic acid inhibits human osteoclast differentiation and bone resorption. PLoS One. 19(10):e0312169. doi:10.1371/journal.pone.0312169. 39405281 PMC11478830

[CIT0031] Pan C, Cheng C, Zhong S, Li S, Tan W, Yao Y. 2025. In vitro study on the promotion of osteogenic differentiation by mitochondrial-derived vesicles through activation of inflammation and reprogramming of metabolic pathways. J Orthop Surg Res. 20(1):388. doi:10.1186/s13018-025-05749-5. 40247396 PMC12007352

[CIT0032] Pappert M, Khosla S, Doolittle M. 2023. Influences of aged bone marrow macrophages on skeletal health and senescence. Curr Osteoporos Rep. 21(6):771–778. doi:10.1007/s11914-023-00820-8. 37688671 PMC10724341

[CIT0033] Quirós PM, Español Y, Acín-Pérez R, Rodríguez F, Bárcena C, Watanabe K, Calvo E, Loureiro M, Fernández-García MS, Fueyo A, et al. 2014. ATP-dependent Lon protease controls tumor bioenergetics by reprogramming mitochondrial activity. Cell Rep. 8(2):542–556. doi:10.1016/j.celrep.2014.06.018. 25017063

[CIT0034] Raghubeer S, Nagiah S, Phulukdaree A, Chuturgoon A. 2015. The phytoalexin resveratrol ameliorates ochratoxin A toxicity in human embryonic kidney (HEK293) cells. J Cell Biochem. 116(12):2947–2955. doi:10.1002/jcb.25242. 26095584

[CIT0035] Reid IR, Billington EO. 2022. Drug therapy for osteoporosis in older adults. Lancet. 399(10329):1080–1092. doi:10.1016/S0140-6736(21)02646-5. 35279261

[CIT0036] Shen L, Yu Y, Zhou Y, Pruett-Miller SM, Zhang GF, Karner CM. 2022. SLC38A2 provides proline to fulfill unique synthetic demands arising during osteoblast differentiation and bone formation. eLife. 11:e76963. doi:10.7554/eLife.76963. PMC9007586 35261338

[CIT0037] Sözen T, Özışık L, Başaran N. 2017. An overview and management of osteoporosis. Eur J Rheumatol. 4(1):46–56. doi:10.5152/eurjrheum.2016.048. 28293453 PMC5335887

[CIT0038] Su H, Yan B, Wang R, Li Z, Xu Z, Xue H, Tan G. 2024. Proteomic analysis based on TMT regarding the therapeutic action of rhizoma drynariae on rats in an osteoporosis model. Comb Chem High Throughput Screen. 27(15):2223–2238. doi:10.2174/0113862073261905231110061401. 38099525 PMC11348476

[CIT0039] Thiyagarajan R, Gonzalez MR, Zaw C, Seldeen KL, Hernandez M, Pang M, Troen BR. 2023. SRT2183 and SRT1720, but not resveratrol, inhibit osteoclast formation and resorption in the presence or absence of Sirt1. J Bone Res. 11(4):1000235. PMC10500633 37711761

[CIT0040] Tosun B, Wolff LI, Houben A, Nutt S, Hartmann C. 2022. Osteoclasts and macrophages-their role in bone marrow cavity formation during mouse embryonic development. J Bone Miner Res. 37(9):1761–1774. doi:10.1002/jbmr.4629. 35689447

[CIT0041] Venkatesh S, Li M, Saito T, Tong M, Rashed E, Mareedu S, Zhai P, Bárcena C, López-Otín C, Yehia G, et al. 2019. Mitochondrial LonP1 protects cardiomyocytes from ischemia/reperfusion injury in vivo. J Mol Cell Cardiol. 128:38–50. doi:10.1016/j.yjmcc.2018.12.017. 30625302

[CIT0042] Videla LA, Marimán A, Ramos B, José Silva M, Del Campo A. 2022. Standpoints in mitochondrial dysfunction: underlying mechanisms in search of therapeutic strategies. Mitochondrion. 63:9–22. doi:10.1016/j.mito.2021.12.006. 34990812

[CIT0043] Wang D, Liu Y, Diao S, Shan L, Zhou J. 2023. Long non-coding RNAs within macrophage-derived exosomes promote BMSC osteogenesis in a bone fracture rat model. Int J Nanomed. 18:1063–1083. doi:10.2147/IJN.S398446. PMC9985426 36879890

[CIT0044] Wang L, Pan Y, Liu M, Sun J, Yun L, Tu P, Wu C, Yu Z, Han Z, Li M, et al. 2023. Wen-Shen-Tong-Luo-Zhi-Tong Decoction regulates bone-fat balance in osteoporosis by adipocyte-derived exosomes. Pharm Biol. 61(1):568–580. doi:10.1080/13880209.2023.2190773. 36999351 PMC10071966

[CIT0045] Wang ZX, Lin X, Cao J, Liu YW, Luo ZW, Rao SS, Wang Q, Wang YY, Chen CY, Zhu GQ, et al. 2024. Young osteocyte-derived extracellular vesicles facilitate osteogenesis by transferring tropomyosin-1. J Nanobiotechnology. 22(1):208. doi:10.1186/s12951-024-02367-x. 38664789 PMC11046877

[CIT0046] Yang N, Liu Y. 2021. The role of the immune microenvironment in bone regeneration. Int J Med Sci. 18(16):3697–3707. doi:10.7150/ijms.61080. 34790042 PMC8579305

[CIT0047] Yilmaz M, Taninmis H, Kara E, Ozagari A, Unsal A. 2012. Nephrotic syndrome after oral bisphosphonate (alendronate) administration in a patient with osteoporosis. Osteoporos Int. 23(7):2059–2062. doi:10.1007/s00198-011-1836-2. 22278748

[CIT0048] Zanini G, Selleri V, Malerba M, Solodka K, Sinigaglia G, Nasi M, Mattioli AV, Pinti M. 2023. The role of Lonp1 on mitochondrial functions during cardiovascular and muscular diseases. Antioxidants. 12(3):598. doi:10.3390/antiox12030598. PMC10045650 36978846

[CIT0049] Zheng S, Ma Y, Guo Y, Dong W, Fan J, Huang G. 2016. Curative effect observation and prescription optimization of “Wenshen Tongluo Zhitong prescription” in the treatment of primary osteoporosis. Liaoning J Trad Chin Med. 43(10):2098–2100.

[CIT0050] Zhou X, Ma Q, Guo Y, Ma Y. 2020. Clinical analysis of the treatment of osteoporosis with the Wen-Shen-Tong-Luo-Zhi-Tong Decoction. Healthc China Abroad. 39(30):157–160 + 166.

[CIT0051] Zhu W, Tan C, Zhang J. 2024. Aging of alveolar type 2 cells induced by Lonp1 deficiency exacerbates pulmonary fibrosis. Biomol Biomed. 24(5):1258–1272. doi:10.17305/bb.2024.10429. 38625722 PMC11378998

