# Supplementary material for: Wen-Shen-Tong-Luo-Zhi-Tong Decoction alleviates bone loss in aged mice by suppressing LONP1-mediated macrophage senescence
Source: Pharm Biol. 2025 Jul 28;63(1):524–48. doi: 10.1080/13880209.2025.2537125 (PMC12305870; doi:10.1080/13880209.2025.2537125)
Supplement: Figure S2.docx [file IPHB_A_2537125_SM5728.docx]

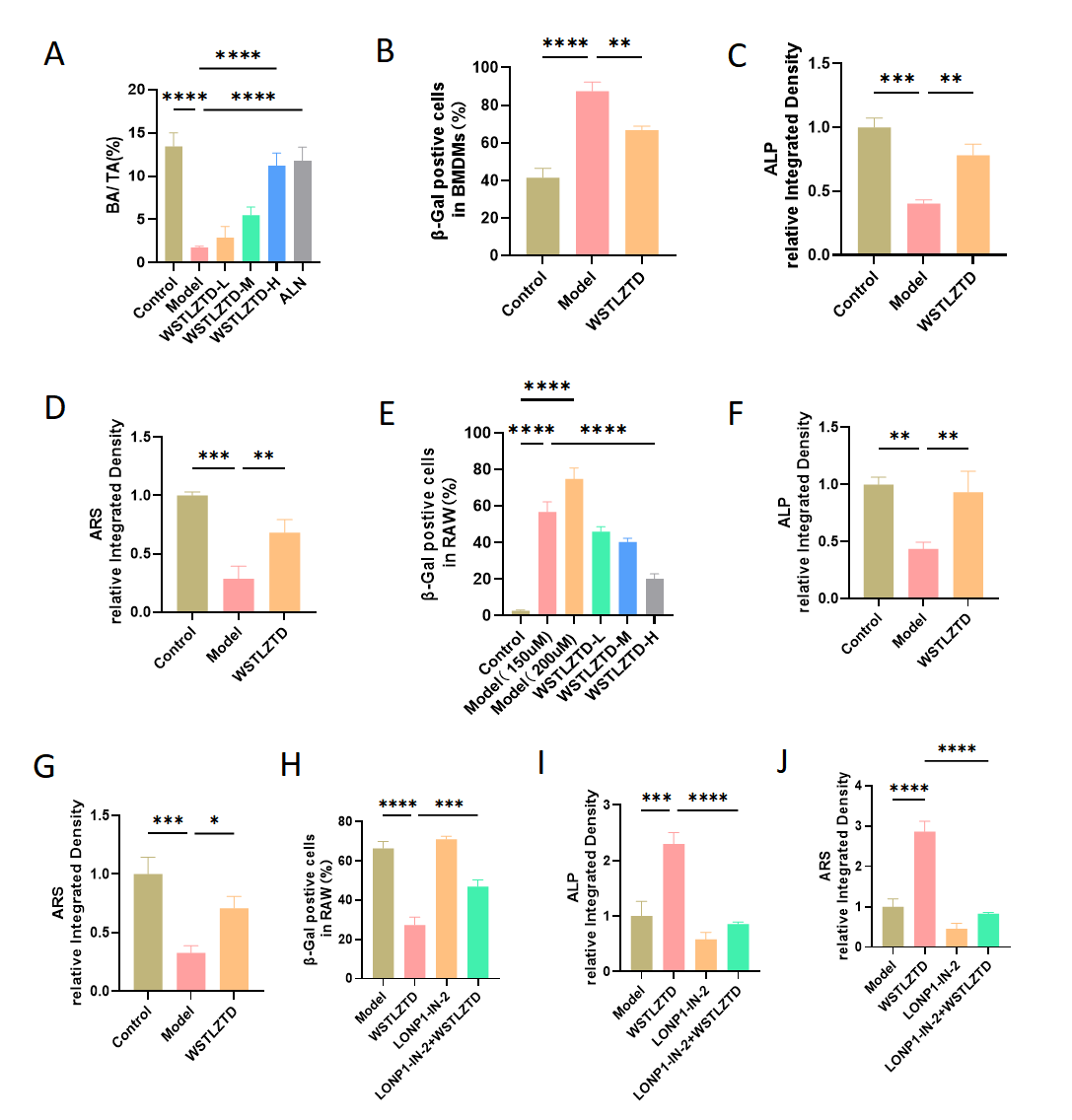


**Supplementary Figure S2. Quantitative analysis for Hematoxylin and Eosin (H&E) staining, β-Galactosidase (β-Gal) staining, ALP staining, and ARS staining.**

A. Quantitative analysis of the proportion of trabecular bone area in the total tissue area in the femur. B.β-Gal positive cells in BMDMs. C. Quantitative analysis of ALP relative Integrated Density. D. Quantitative analysis of ARS relative Integrated Density. E.β-Gal postive cells in RAW 264.7 cells. F. Quantitative analysis of ALP relative Integrated Density. G. Quantitative analysis of ARS relative Integrated Density. H.β-Gal positive cells in RAW 264.7 cells. I.Quantitative analysis of ALP relative Integrated Density. J. Quantitative analysis of ARS relative Integrated Density.
