# Supplementary material for: Wen-Shen-Tong-Luo-Zhi-Tong Decoction alleviates bone loss in aged mice by suppressing LONP1-mediated macrophage senescence
Source: Pharm Biol. 2025 Jul 28;63(1):524–48. doi: 10.1080/13880209.2025.2537125 (PMC12305870; doi:10.1080/13880209.2025.2537125)
Supplement: Figure S1.docx [file IPHB_A_2537125_SM5727.docx]

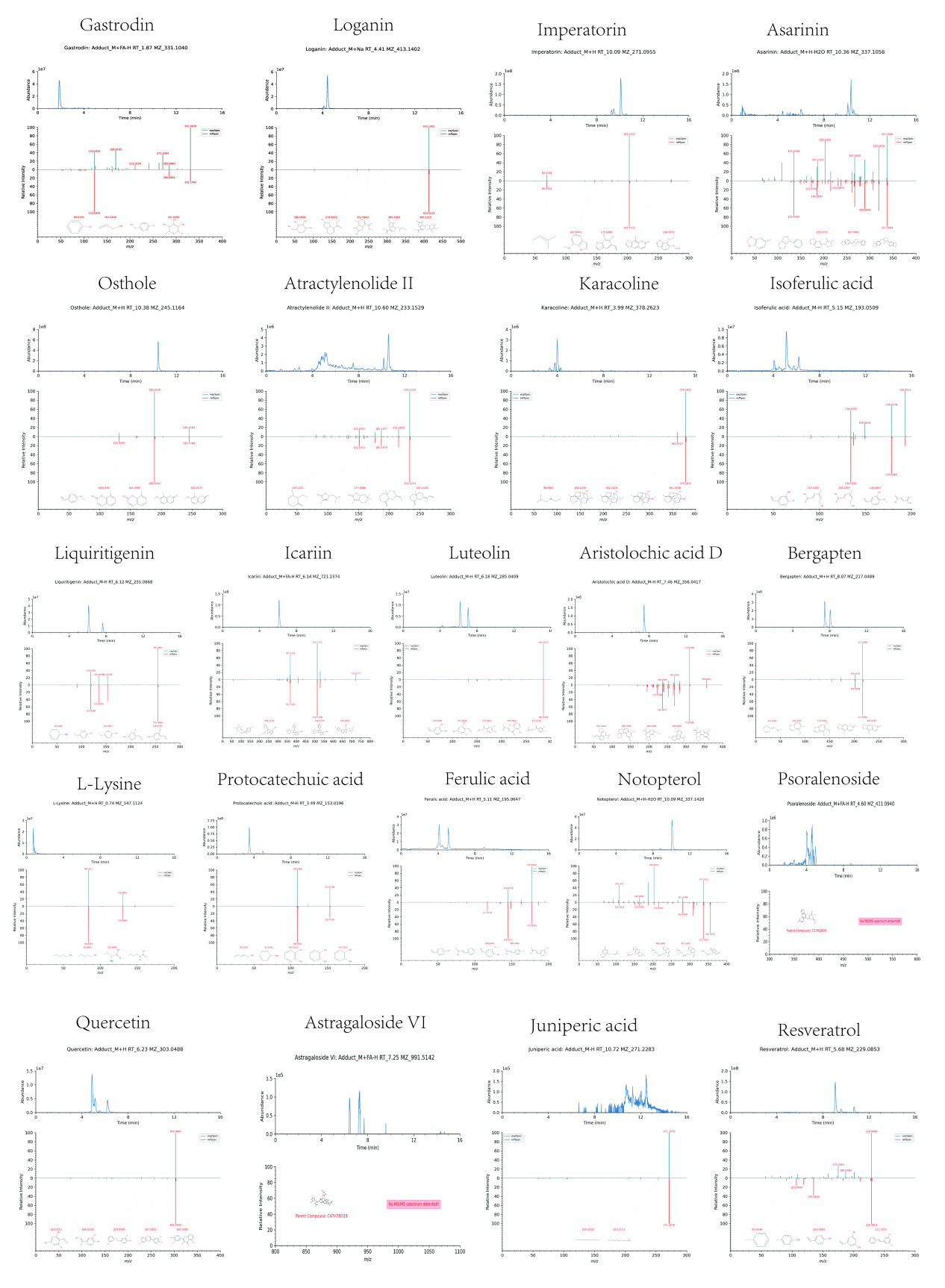


**Supplementary Figure S1. EICs and secondary mass spectrometry of main bioactive compounds.**

Supplementary Figure S1 presents the extracted ion chromatograms (EICs) and secondary mass spectrometry data for the principal bioactive compounds identified in the analysis depicted in Figure 1A.
